# Freshness‐Dependent Effects of Degreased *Hermetia illucens* Meal as a Fishmeal Substitute on Growth Performance, Edible Muscle Nutritional Quality, and Physiological Status of *Macrobrachium nipponense*

**DOI:** 10.1002/fsn3.72274

**Published:** 2026-09-07

**Authors:** Huang Xin, Xue Bin, Wei Shen, Wu Guo Hua, Huang Yangdi, Li Danyang, Jing Sha, Yang Leyan, Xing Er Luo, Sheng Yin Wang

**Affiliations:** ^1^ Xingzhi College, Zhejiang Normal University Jinhua Zhejiang China; ^2^ College of Advanced Agricultural Sciences Zhejiang A&F University Hangzhou Zhejiang China; ^3^ Science and Technology Bureau of Nanhu District Jiaxing Zhejiang China; ^4^ Zhejiang Kaiyue Biotechnology Co. Ltd. Jiaxing Zhejiang China

**Keywords:** black soldier fly, growth performance, *Hermetia illucens*, *Macrobrachium nipponense*, substitution ratio

## Abstract

Degreased *Hermetia illucens* (black soldier fly) meal has been widely used as a substitute for traditional protein sources, including fish and soybean meals, in livestock and aquaculture especially for prawn *Litopenaeus vannamei* and *Macrobrachium rosenbergii* . The oriental river prawn ( *Macrobrachium nipponense* ) is a commercially significant shrimp species prevalent in freshwater lakes and river systems across China. We explored the potential of replacing fish meal with degreased *H. illucens* meal in the diet of *M. nipponense* by assessing how different substitution ratios and the freshness of the meal influenced river prawn growth. The results showed that a 20% substitution ratio significantly increased the specific growth rate and condition factor of *M. nipponense* (*p* < 0.05), while the feed conversion ratio was significantly reduced (*p* < 0.05). Based on the dose–response model, the specific growth rate reaches its peak when the substitution ratio is 24.25%. At this 20% ratio, there was a significant increase in the crude protein and lipid content in *M. nipponense* muscle. Moreover, the concentrations of several essential amino acids, along with monounsaturated and polyunsaturated fatty acids, were notably elevated compared to the control group (*p* < 0.05). In the 20% substitution group, the activities of superoxide dismutase, trypsin, and lipase in the hepatopancreas, as well as acid phosphatase and lysozyme in the hemolymph, were significantly increased relative to the control group (*p* < 0.05). The research also assessed the impact of using moderately decomposed (T_1_: TVB‐N 65 mg/100 g) and highly decomposed (T_2_: TVB‐N 80 mg/100 g) degreased *H. illucens* meal as a 20% replacement for fish meal, in comparison to fresh degreased *H. illucens* meal (TVB‐N 50 mg/100 g). In the T_1_ group, both the specific growth rate and feed conversion ratio were considerably lower than those observed in the control group (*p* < 0.05). In both the moderately (T_1_) and highly (T_2_) decomposed treatment groups, the crude lipid content in *M. nipponense* muscle was significantly reduced, while the crude ash content was notably higher than that in the control group (*p* < 0.05). Additionally, the T_1_ and T_2_ groups exhibited significantly decreased levels of certain essential amino acids, saturated fatty acids, monounsaturated fatty acids, polyunsaturated fatty acids, and mineral elements in the muscle (*p* < 0.05). In these groups, the activities of alanine aminotransferase in the hepatopancreas and hemolymph, as well as the malondialdehyde content in the hepatopancreas, were significantly elevated compared to the control group (*p* < 0.05). On the other hand, the activities of hepatopancreatic trypsin and lipase in the T_2_ treatment were significantly reduced compared to the control group (*p* < 0.05). In conclusion, we recommend the use of fresh degreased *H. illucens* meal at a substitution level of 20%–25% (with the model‐derived peak near 24.25%) in *M. nipponense* diets.

## Graphical abstract

*Hermetia illucens* (black soldier fly) is a saprophagous resource insect that is rich in protein and fat. Replacing 20% of fishmeal with degreased *H. illucens* meal could significantly improve the growth performance and nutrient utilization of *Macrobrachium nipponense* (oriental river prawn). However, the moderately decomposed and highly decomposed degreased *H. illucens* meal significantly decreased the growth performance of the prawn and adversely affects its digestive enzyme activities and immune function. Therefore, using fresh degreased *H. illucens* meal as a substitute for fishmeal in feed production for *M. nipponense* represents a sustainable and cost‐effective alternative protein source.

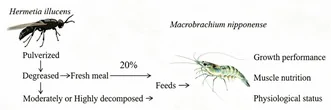

## 1 Introduction

Chronic overfishing has resulted in a persistent decline in global marine fishery resources. Concurrently, the global aquaculture industry encounters dual challenges: the scarcity of fish meal and associated feed safety risks. These issues have contributed to a continuous increase in the cost of fish farming and significant uncertainty regarding the sustainability of fishmeal supply (Zubair 2026). *Hermetia illucens* (black soldier fly) is an insect resource with outstanding advantages, including rapid growth, high feed conversion rate, and high protein content (Mkilima et al. 2026). It can efficiently convert low‐value agricultural organic waste into high‐value insect protein for use as an environmentally friendly, novel protein feed (Zoli et al. 2026). Degreased *H. illucens* meal is currently extensively utilized in the aquaculture of various species, such as rainbow trout ( *Oncorhynchus mykiss* ), yellow catfish ( *Pelteobagrus fulvidraco* ), large yellow croaker ( *Larimichthys crocea* ), snakehead ( *Channa argus* ), mandarin fish ( *Siniperca chuatsi* ), largemouth bass ( *Micropterus salmoides* ), giant freshwater prawn ( *Macrobrachium rosenbergii* ), and *Procambarus clarkii* (Chen et al. 2025; Han et al. 2023; Zhang, Liu, et al. 2024; Wang, Xu, et al. 2026; Wang, Ding, et al. 2026). Using *H. illucens* to convert agricultural organic waste into degreased meal to replace fish meal can not only reduce dependence on imported fish meal protein and alleviate marine ecological pressure, but also significantly lower feed costs and the carbon footprint in aquaculture (Galán Díaz et al. 2026). However, it is still unclear whether the degreased *H. illucens* meal can be used for breeding *Macrobrachium nipponense* .

The quality of degreased *H. illucens* meal can be affected by degreasing processing, storage conditions, and transportation speed, which may lead to protein degradation and spoilage, further impacting the freshness and palatability of aquafeeds (Chen et al. 2025; Zhang et al. 2026). Numerous studies have demonstrated that the quality of protein sources, particularly fish meal, plays a significant role in influencing the feed utilization efficiency and growth performance of aquatic animals. This has been observed in species such as Atlantic wolffish ( *Anarhichas lupus* ) (Moksness et al. 2008), Atlantic halibut ( *Hippoglossus hippoglossus* ) (Aksnes and Mundheim 1997), Atlantic cod ( *Gadus morhua* ) (Albrektsen et al. 2006), and *Litopenaeus vannamei* (Tantikitti et al. 2016). Consequently, maintaining high‐quality protein sources during feed production is crucial for optimizing the production performance and nutrient content of *M. nipponense* .

Total volatile basic nitrogen (TVB‐N) is commonly used to assess the freshness of protein meals added to animal feed (Bekhit, Holman, et al. 2021). During the spoilage of fish meal and degreased *H. illucens* meal, proteins decompose into malodorous compounds such as ammonia, amines, and other basic nitrogenous substances (Chen et al. 2019; Servillo et al. 2018). The concentration and content of TVB‐N influence not only the palatability of feed for aquatic animals but also disrupt the amino acid balance within the feed, thereby diminishing the animals' efficiency in amino acid utilization (Bekhit, Giteru, et al. 2021). Ricque‐Marie et al. (1998) observed that an increase in the TVB‐N value of decomposed fish meal to 50 mg/100 g significantly decreased the growth performance parameters of *L. vannamei* . Lin et al. (2021) demonstrated that storing fish meal at room temperature for 135 days elevated its TVB‐N value to 61.41 mg/100 g, which significantly reduced the activities of hepatic glutathione peroxidase and superoxide dismutase in juvenile grouper ( *Epinephelus coioides* ). Xu et al. (2024) showed that the weight gain rate (WGR) and specific growth rate (SGR) of *M. rosenbergii* significantly decreased with the feed TVB‐N value increased. Wu et al. (2025) reported that feeds formulated with moderately and highly decomposed fish meal, resulting in dietary TVB‐N values of 65 and 80 mg/100 g, had significant adverse effects on the growth performance and superoxide dismutase activity of *M. rosenbergii* , and induced extensive hepatopancreatic cell death. Wang, Xu, et al. (2026); Wang, Ding, et al. (2026) indicated that DT65 (dietary TVB‐*N* = 65 mg/100 g) and DT76 (dietary TVBN = 80 mg/100 g) feed significantly impaired growth performance of *M. rosenbergii* and increased the feed conversion ratio (FCR). In most regions of China, the *M. nipponense* culture period (March to November) spans the hot and humid summer months (Jiang et al. 2025; Liu, Hu, et al. 2025), during which proteins in the feed are prone to spoilage, increasing the TVB‐N content and potentially reducing prawn growth performance and nutrient content. However, the effect of freshness of degreased *H. illucens* meal on the growth and nutrition of *M. nipponense* remains to be explored.

The oriental river prawn, *M. nipponense* , commonly known as river or freshwater shrimp in China, is a widely distributed edible species found in the freshwater lakes and river systems of the country (Li, Xie, et al. 2024; Liu, Hu, et al. 2025; Liu, Mo, et al. 2025). It is naturally distributed in most East Asian countries, including China, Korea, Japan, Vietnam, Myanmar, Cambodia, Thailand, Malaysia, and Singapore (Zhang, Zhou, et al. 2024). With the increasing consumer demand for *M. nipponense* in China, research on its culture techniques has gained significant attention. Worlanyo et al. (2022) found that a significant increasing trend of final body weight, specific growth rate, protein efficiency ratio, and weight gain rate of oriental river prawn ( *M. nipponense* ) occurs when threonine levels increase to 1.67%. According to Li, Liu, et al. (2023), incorporating 25% fermented feed can boost the rate of weight gain, strengthen antioxidant capacity, and enhance the diversity of intestinal microbiota in *M. nipponense* . Qian et al. (2023) indicated that supplementing the feed with 165.25–180.31 mg/kg can enhance the growth performance and feed efficiency of *M. nipponense* , while also significantly upregulating processes like glycolysis, fatty acid β‐oxidation, and protein synthesis. Ettefaghdoost and Haghighi (2025) found that the incorporation of 4 g/kg of chitosan into the diet of *M. nipponense* has a beneficial effect on their growth, hematological, immuno‐physiological, and metabolic parameters. These findings provide technical guidance for building a nutrient requirement database and developing efficient feed formulations for *M. nipponense* . Exploring the proportion of degreased *H. illucens* meal replacing fish meal and the effect of meal freshness on the growth and nutrient content of *M. nipponense* can help expand the shrimp aquaculture industry.

Degreased *H. illucens* meal is widely used in aquaculture as a fish meal substitute to reduce production costs. Although *M. nipponense* is a popular freshwater shrimp in the Chinese market, no studies have reported the use of degreased *H. illucens* meal in its diet. To establish a green circular agricultural production technology system for feeding *M. nipponense* with degreased *H. illucens* meal as a fish meal substitute, this study aimed to assess the impact of both the substitution ratio and the freshness of degreased *H. illucens* meal on the culture of *M. nipponense* .

## 2 Materials and Methods

### 2.1 Experimental Prawns

Juvenile *M. nipponense* (about 1000 tails/kg) specimens were procured from Zhejiang Wuyue Agricultural Co. Ltd. Upon their arrival at the laboratory, the juvenile prawns underwent a one‐week observation period in a quarantine tank (2 m × 3 m in size) to reduce environmental stress. Weak, diseased, or injured individuals were excluded. Healthy, uninjured prawns of uniform length and similar body weights were selected for the experiment.

### 2.2 Feed Formulation

#### 2.2.1 Feed Formulation for Substitution Ratio Experiment

In the experimental diets, fish meal and degreased *Hermetia illucens* meal were employed as protein sources. Lipid sources included fish and soybean oils, while pregelatinized starch was utilized as the primary carbohydrate source. The degreased *H. illucens* meal was substituted for fish meal at ratios of 10% (T_10_), 20% (T_20_), 30% (T_30_), 40% (T_40_), and 50% (T_50_). The control group (CK) did not contain any degreased *H. illucens* meal. All ingredients were thoroughly mixed, mechanically pulverized, and sieved through an 80‐mesh to eliminate larger particles. Subsequently, the mixture was processed into pellets with a diameter of 1.5 mm using a feed pelletizer (model KYW32a). The pellets were freeze‐dried and stored in sealed bags at −20°C (Haier BCD‐577) until required (Table 1). The feed formulation adhered to the principles of being isonitrogenous, isolipidic, and isoenergetic.

**TABLE 1:** Ingredient and proximate composition of experimental diets (dry matter, %).

	CK	T_10_	T_20_	T_30_	T_40_	T_50_
Ingredients						
Fish meal	58.0	52.2	46.4	40.6	34.8	29.0
Degreased *H. illucens* meal	—	5.8	11.6	17.4	23.2	29.0
Pregelatinized starch	26.0	26.0	26.0	26.0	26.0	26.0
Fish oil:soybean oil (2:1)	4.0	4.0	4.0	4.0	4.0	4.0
Soybean lecithin	0.5	0.5	0.5	0.5	0.5	0.5
Cholesterol	0.5	0.5	0.5	0.5	0.5	0.5
Choline chloride	0.5	0.5	0.5	0.5	0.5	0.5
Vitamin premix 1	2.0	2.0	2.0	2.0	2.0	2.0
Mineral premix 2	3.0	3.0	3.0	3.0	3.0	3.0
Cellulose	3.5	3.5	3.5	3.5	3.5	3.5
Carboxymethylcellulose sodium	2.0	2.0	2.0	2.0	2.0	2.0
Total	100	100	100	100	100	100
Proximate composition (dry matter)						
Moisture	4.4	4.4	4.4	4.4	4.4	4.4
Crude protein	42.4	42.4	42.5	42.7	42.8	42.8
Crude lipid	8.3	8.3	8.3	8.3	8.3	8.3
Crude ash	6.6	6.6	6.5	6.4	6.3	6.3
Nitrogen‐free extract 3	38.3	38.3	38.3	38.2	38.2	38.2
Gross energy	19.3	19.3	19.3	19.3	19.3	19.3

*Note:* (1) The vitamin premix composition per kilogram included: VA 1.29 g, VC 57.5 g, VE 20 g, VD3 0.63 g, VK3 1.8 g, VB1 7.5 g, VB2 2.63 g, VB6 1 g, VB12 0.15 g, nicotinic acid 5 g, folic acid 0.19 g, inositol 60 g, biotin 0.75 g, calcium pantothenate 5 g, aminobenzoic acid 5 g, and α‐cellulose 831.56 g. (2) The mineral premix composition per kilogram included: KCl 28 g, MgSO_4_·7H_2_O 100 g, NaH_2_PO_4_ 215 g, KH_2_PO_4_ 100 g, Ca(H_2_PO_4_)2·H_2_O 265 g, CaCO_3_ 105 g, C_6_H_10_CaO_6_·5H_2_O 165 g, FeC_6_H_5_O_7_·5H_2_O 12 g, ZnSO_4_·7H2O 4.76 g, MnSO_4_·H2O 1.07 g, AlCl_3_·6H_2_O 0.15 g, CuCl_2_·2H_2_O 2.4 g, CoCl_2_·6H_2_O 1.4 g, KI 0.23 g, and α‐cellulose 0.043 g. (3) The nitrogen‐free extract is calculated as dry matter percentage minus the sum of crude protein percentage, crude lipid percentage, crude fiber percentage, and crude ash percentage. (4) Gross energy is determined by the formula: (crude protein percentage × 16.7 kJ/g + crude lipid percentage × 37.6 kJ/g + nitrogen‐free extract percentage × 16.7 kJ/g) divided by dry matter percentage.

#### 2.2.2 Feed Formulation for Freshness Experiment

The protein sources were fish meal and degreased *H. illucens* meal, with lipids from fish oil and soybean oil and pre‐gelatinized starch as the carbohydrate source (Table 2). Degreased *H. illucens* meal was mixed with water and incubated in a biochemical incubator (JC100‐A, Qingdao Jingcheng Instrument Co. Ltd.) at 25°C for 24 and 48 h. The TVB‐N value was measured every 4 h using a Kjeldahl nitrogen analyzer (JC‐NY8B, Qingdao Jingcheng Instrument Co. Ltd.). The TVB‐N values for fish meal were 54.77 mg/100 g, and the TVB‐N values for fresh, moderately decomposed, and highly decomposed degreased *H. illucens* meal were 30.93, 159.80, and 331.62 mg/100 g, respectively. Subsequently, 20% fresh, 20% moderately decomposed, or 20% highly decomposed degreased *H. illucens* meal was added to a basal feed mixture. TVB‐N value = (fish meal weight × 54.77 + degreased *H. illucens* meal weight × TVB‐N value)/(fish meal weight + degreased *H. illucens* meal weight). The resulting TVB‐N values for the three formulated diets were 50 mg/100 g (CK), 65 mg/100 g (T_1_), and 80 mg/100 g (T_2_), respectively. The ingredients were mixed, pelleted into 1.5 mm diameter pellets (KYW32a), freeze‐dried, and stored at −20°C (Haier BCD‐577). After refrigeration, the TVB‐N value remains unchanged. In the feed formula, the principles of isonitrogenous, isolipidic, and isoenergetic have been basically achieved.

**TABLE 2:** Ingredient and proximate composition of experimental diets (dry matter, %).

	CK	T_1_	T_2_
Ingredients			
Fish meal	46.4	46.4	46.4
Fresh degreased *H. illucens* meal	11.6		
Moderately decomposed degreased *H. illucens* meal		11.6	
Highly decomposed degreased *H. illucens* meal			11.6
Pregelatinized starch	26.0	26.0	26.0
Fish oil:soybean oil (2:1)	4.0	4.0	4.0
Soybean lecithin	0.5	0.5	0.5
Cholesterol	0.5	0.5	0.5
Choline chloride	0.5	0.5	0.5
Vitamin premix 1	2.0	2.0	2.0
Mineral premix 2	3.0	3.0	3.0
Cellulose	3.5	3.5	3.5
Carboxymethylcellulose sodium	2.0	2.0	2.0
Proximate composition (dry matter)	100	100	100
Moisture	4.4	4.4	4.4
Crude protein	42.4	42.4	42.5
Crude lipid	8.3	8.3	8.3
Crude ash	6.6	6.6	6.5
Nitrogen‐free extract 3	38.3	38.3	38.3
Gross energy	19.3	19.3	19.3

*Note:* The vitamin premix, mineral premix, nitrogen‐free, and gross energy are the same as in Table 1.

### 2.3 Culture Management

#### 2.3.1 Effect of Substitution Ratio

The experiment was conducted in square concrete ponds (2 m × 3 m in size). The pond bottoms were covered with 5 cm of sand to simulate the natural environment and help maintain the water quality. Ponds and tools were disinfected by soaking in potassium permanganate for 48 h, followed by rinsing and daily water changes for 7 days. Water depth was 1.2 m. Multi‐layered porous concrete blocks were placed along the pond edges as shelters to reduce cannibalism in the prawn population. Treatments T_10_, T_20_, T_30_, T_40_, and T_50_, and the control (CK) each had five replicate ponds stocked with 1000 juveniles per pond. The prawns were fed twice daily at 8:00 and 18:00, in accordance with commercial practices. The feed was utilized within 2 h of being removed from the −20°C freezer to prevent spoilage. Daily monitoring was conducted for water temperature, dissolved oxygen, ammonia, and nitrate levels. Approximately 30% of the water was exchanged each day, and all of the dead individuals were also removed as soon as possible. The trial spanned 120 days.

#### 2.3.2 Effect of Freshness of Degreased *H. illucens* Meal

The experimental design included three groups: 20% fresh degreased *H. illucens* meal (CK), 20% moderately decomposed degreased *H. illucens* meal (T_1_), and 20% highly decomposed degreased *H. illucens* meal (T_2_). Each group had five replicate ponds, each stocked with 1000 *M. nipponense* juveniles. The culture methods were the same as those described in Section 2.3.1.

### 2.4 Sample Collection and Growth Performance Measurement

Upon conclusion of the trial, prawns underwent a 24‐h starvation period. Subsequently, all individuals in each pond were harvested and counted to determine the survival rate. Additionally, the prawns in each pond were weighed. The growth performance indices were calculated as follows:

1. Survival rate (%): $\mathrm{SR}=\frac{n_{2}}{n_{1}}\times 100$
2. Weight gain (%): $\mathrm{WG}=\frac{W_{t}-W_{0}}{W_{0}}\times 100.$
3. Specific growth rate (%): $\mathrm{SGR}=\frac{\ln W_{t}-\ln W_{0}}{t}\times 100$
4. Feed conversion ratio (%): $\mathrm{FCR}=\frac{F}{W_{t}-W_{0}}\times 100$

Where: *W*_0_ = initial body weight (g), *W*_t_ = final body weight (g), *F* = feed intake (g), *t* = culture period (days), *n*_1_ = initial number, *n*_2_ = final number.

Based on the substitution ratio of degreased *H. illucens* meal for fish meal and the specific growth rate of *M. nipponense* , a concentration response model was constructed to calculate the optimal substitution ratio.

### 2.5 Determination of Shrimp Body Composition

Twenty live prawns, comprising 10 males and 10 females, were randomly selected from each treatment and control group. The abdominal muscles were dissected, freeze‐dried to a constant weight, and subsequently analyzed. The crude protein, crude lipid, crude ash, and moisture content were determined using the methodologies outlined by Zhu et al. (2022) and Jiang et al. (2025). The amino acid composition was analyzed with a fully automatic amino acid analyzer (L‐8900) in accordance with the GB/T5009.124‐2003 standard. The fatty acid composition was assessed using high‐performance gas chromatography (EXPEC 3700, Hangzhou Puyu Technology Development Co. Ltd.) following the GB/T5009.168‐2016 standard (Zheng et al. 2022). The mineral element content was determined using the methods described by Tang et al. (2021).

### 2.6 Determination of Immune and Antioxidant Indices

The hepatopancreas was precisely dissected and weighed (0.5 g). Pre‐chilled (15 min) 0.86% saline was added at a 1:9 (w/v) ratio for homogenization. The protein content in the homogenate was determined using the Coomassie Brilliant Blue method. The activities of aspartate aminotransferase (AST), alanine aminotransferase (ALT), superoxide dismutase (SOD), glutathione peroxidase (GSH‐Px), and malondialdehyde (MDA) were measured using ELISA kits (Nanjing Jiancheng Bioengineering Institute).

Hemolymph was collected from the ventral sinus of prawns from each group using a sterile syringe. It was mixed 1:1 with anticoagulant, placed in an ice‐water bath for 1 h, and then refrigerated at 4°C for more than 12 h. Following clotting, the mixture was centrifuged at 3000 rpm for 15 min at low temperature, and the supernatant was collected for further analysis. The supernatant was used to measure AST, ALT, acid phosphatase (ACP), alkaline phosphatase (AKP), and lysozyme (LZM) activities using ELISA kits (Nanjing Jiancheng Bioengineering Institute, Nanjing, China).

### 2.7 Data Analysis

All data are presented as the mean ± standard error (SE) based on independent biological replicates, with each biological replicate representing a pond. Statistical analyses were conducted using the Statistical Package for the Social Sciences 22.0 software (SPSS Inc., Chicago, IL, USA). Significant differences among multiple samples were determined using one‐way ANOVA followed by the Tukey test, with means separated at the level of *p* < 0.05.

## 3 Results and Analysis

### 3.1 Growth Performance of *M. nipponense*

**FIGURE 1:**
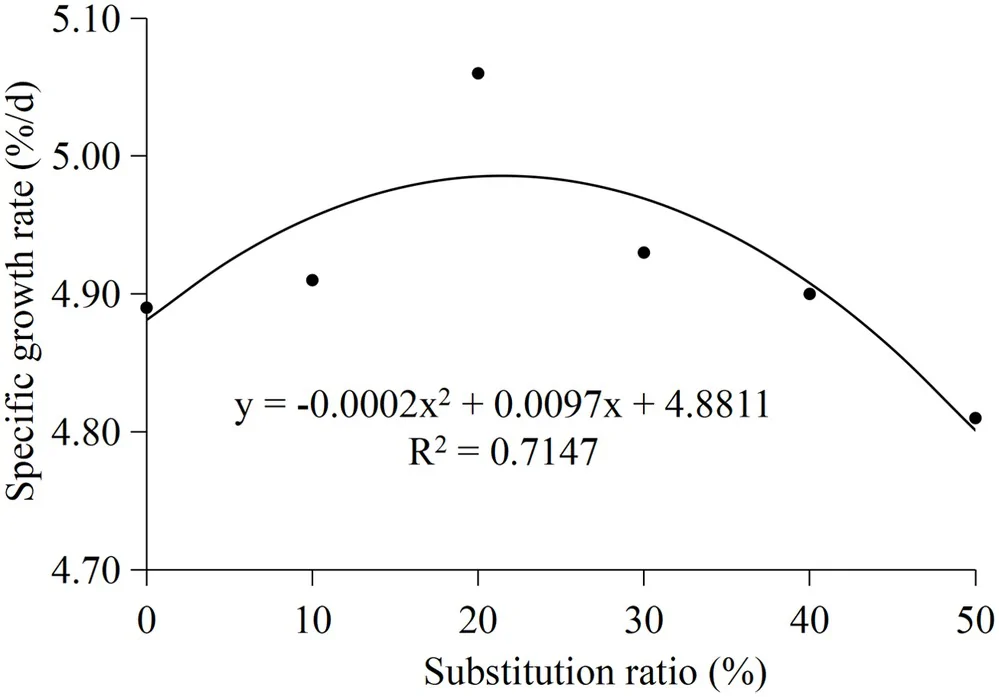
Dose–response model of the specific growth rate of *Macrobrachium nipponense* and substitution ratio of degreased *Hermetia illucens* meal for fish meal.

The partial replacement of fish meal with degreased *H. illucens* meal had a significant impact on the growth performance of *M. nipponense* , as shown in Table 3. Specifically, when degreased *H. illucens* meal constituted 20% of the fish meal, the final body weight, weight gain rate, specific growth rate, condition factor, and survival rate were all significantly higher compared to the control group (*p* < 0.05). Additionally, the weight gain rate, specific growth rate, condition factor, and survival rate in the T_50_ group were significantly lower than that of the control group (*p* < 0.05). Based on the dose–response model of the specific growth rate of *M. nipponense* and the substitution ratio of degreased *H. illucens* meal for fish meal (*y* = −0.0002*x* ^2^ + 0.0097*x* + 4.8811, *R* ^2^ = 0.7147), the theoretical maximum specific growth rate (SGR) is predicted to occur at a substitution ratio of approximately 24.25% (Figure 1). Notably, this value falls within the range of the experimentally tested 20% and 30% groups. Given the moderate *R* ^2^ value and the lack of direct testing at 24.25%, the optimal substitution level is likely between 20% and 25%, rather than at a single fixed point.

**TABLE 3:** Effect of the substitution ratio of degreased *Hermetia illucens* meal with fish meal on the growth performance of *Macrobrachium nipponense* .

Growth indicators	CK	T_10_	T_20_	T_30_	T_40_	T_50_	
Initial body weight (IBW), g/pond	21.79 ± 0.72a	22.53 ± 1.05a	22.66 ± 0.56a	22.92 ± 0.42a	21.99 ± 1.01a	23.21 ± 0.75a	*F*_5,24_ = 0.474, *p* < 0.05
Final body weight (FBW), g/pond	7571.24 ± 315.68b	7992.53 ± 201.12b	9612.36 ± 481.48a	8363.57 ± 371.82ab	7724.12 ± 212.35b	7724.12 ± 212.35b	*F*_5,24_ = 7.322, *p* < 0.05
Weight gain rate (WGR), %	34876.67 ± 2235.01ab	35578.20 ± 1336.26ab	42554.17 ± 2924.46a	36410.062 ± 1597.32ab	35341.51 ± 2033.32ab	31398.35 ± 960.66b	*F*_5,24_ = 3.461, *p* < 0.05
Specific growth rate (SGR), %/d	4.87 ± 0.05b	4.90 ± 0.03b	5.04 ± 0.06a	4.91 ± 0.04ab	4.89 ± 0.05b	4.79 ± 0.03b	*F*_5,24_ = 3.484, *p* < 0.05
Feed conversion ratio (FCR)	1.72 ± 0.09a	1.60 ± 0.10ab	1.31 ± 0.09b	1.51 ± 0.09ab	1.60 ± 0.04ab	1.70 ± 0.7a	*F*_5,24_ = 3.281, *p* < 0.05
Body length (BL), cm	6.46 ± 0.08a	6.47 ± 0.06a	6.46 ± 0.10a	6.44 ± 0.08a	6.44 ± 0.09a	6.49 ± 0.07a	*F*_5,24_ = 0.062, *p* < 0.05
Condition factor (CF), %	2.83 ± 0.20b	2.95 ± 0.11ab	3.60 ± 0.26a	3.13 ± 0.17ab	2.90 ± 0.15ab	2.66 ± 0.11b	*F*_5,24_ = 3.453, *p* < 0.05
Survival rate (SR), %	55.96 ± 1.34ab	57.80 ± 0.93ab	60.52 ± 1.07a	58.14 ± 1.06ab	55.56 ± 0.93ab	54.78 ± 1.12b	*F*_5,24_ = 3.819, *p* < 0.05

*Note:* Data marked with different lowercase letters in the same column indicate significant differences (Tukey‐B, *p* < 0.05). Results are presented as mean ± standard error (SE). The below is the same.

The freshness of the degreased *H. illucens* meal significantly affected the growth performance of *M. nipponense* (Table 4). There was no significant initial weight difference between the groups. When moderately decomposed (T_1_) or highly decomposed (T_2_) degreased *H. illucens* meal replaced 20% of fish meal, the final body weight, weight gain rate, specific growth rate, and feed conversion ratio were significantly worse (lower for FBW, WG, SGR; higher for FCR) than those of the control (*p* < 0.05). In the T_2_ group, the condition factor and survival rate were significantly lower than those in the control group (*p* < 0.05). Body lengths in T_1_ and T_2_ were not significantly different from those in the control.

**TABLE 4:** Effects of freshness of degreased *Hermetia illucens* meal on growth performance of *Macrobrachium nipponense* .

Growth indicators	CK	T_1_	T_2_	
Initial body weight (IBW), g/pond	22.59 ± 0.52a	23.45 ± 0.64a	23.52 ± 0.42a	*F*_2,12_ = 0.940, *p* = 0.417
Final body weight (FBW), g/pond	8242.67 ± 430.51a	654.89 ± 415.65b	5997.76 ± 199.41b	*F*_2,12_ = 9.677, *p* < 0.05
Weight gain rate (WGR), %	37101.67 ± 1027.92a	29703.26 ± 1730.54b	26220.42 ± 915.78b	*F*_2,12_ = 18.943, *p* < 0.05
Specific growth rate (SGR), %/d	4.93 ± 0.02a	4.74 ± 0.05b	4.64 ± 0.03b	*F*_2,12_ = 17.851, *p* < 0.05
Feed conversion ratio (FCR)	1.63 ± 0.09b	1.99 ± 0.12a	2.29 ± 0.06a	*F*_2,12_ = 12.903, *p* < 0.05
Body length (BL), cm	6.45 ± 0.07a	6.36 ± 0.02a	6.29 ± 0.04a	*F*_2,12_ = 2.526, *p* = 0.121
Condition factor (CF), %	3.09 ± 0.23a	2.67 ± 0.19ab	2.40 ± 0.07b	*F*_2,12_ = 3.769, *p* < 0.05
Survival rate (SR), %	57.26 ± 2.11a	53.54 ± 1.50ab	49.60 ± 1.06b	*F*_2,12_ = 5.611, *p* < 0.05

*Note:* Data marked with different lowercase letters in the same column indicate significant differences (Tukey‐B, *p* < 0.05). Results are presented as mean ± standard error (SE).

### 3.2 Nutrient Content of *M. nipponense*

In the analysis of whole shrimp, the crude protein, crude lipid, and crude ash contents in the *H. illucens* substitution groups exceeded those in the control group; however, these differences were not statistically significant (*p* > 0.05). In terms of muscle measurements, the crude protein content in the T_10_ and T_20_ groups was significantly greater than that in the control group (*p* < 0.05). Additionally, the crude ash content in the T_20_, T_30_, and T_40_ groups was significantly higher compared to the control (*p* < 0.05) (Table 5). The crude lipid content in the T_20_ group was significantly higher than that in the T_50_ group (*p* < 0.05), though no significant difference was observed between T_20_ and the control (*p* > 0.05) (Table 5).

**TABLE 5:** Effect of the substitution ratio of degreased *Hermetia illucens* meal with fish meal on nutrient content in *Macrobrachium nipponense* (%).

Groups		CK	T_10_	T_20_	T_30_	T_40_	T_50_	
Whole shrimp	Moisture	78.92 ± 0.92a	77.81 ± 0.95a	77.72 ± 1.04a	77.31 ± 0.77a	77.54 ± 1.23a	77.55 ± 0.73a	*F*_5,24_ = 0.359, *p* = 0.871
	Crude protein	16.74 ± 0.53b	18.05 ± 0.38ab	19.54 ± 0.53a	18.17 ± 0.33ab	17.88 ± 0.42ab	16.81 ± 0.61b	*F*_5,24_ = 4.659, *p* < 0.05
	Crude fat	1.65 ± 0.13a	1.87 ± 0.05a	1.92 ± 0.10a	1.85 ± 0.07a	1.81 ± 0.07a	1.64 ± 0.16a	*F*_5,24_ = 1.320, *p* = 0.289
	Crude ash	1.56 ± 0.05a	1.68 ± 0.06a	1.75 ± 0.02a	1.75 ± 0.04a	1.74 ± 0.07a	1.74 ± 0.03a	*F*_5,24_ = 2.581, *p* = 0.053
Shrimp muscle	Moisture	77.23 ± 0.75ab	74.73 ± 1.17c	74.94 ± 0.50bc	74.91 ± 0.48bc	76.62 ± 0.73abc	77.53 ± 0.66a	*F*_5,24_ = 2.916, *p* < 0.05
	Crude protein	18.87 ± 0.46c	22.06 ± 0.50ab	22.28 ± 0.99a	21.05 ± 0.43abc	19.52 ± 0.93bc	18.76 ± 0.75c	*F*_5,24_ = 6.601, *p* < 0.05
	Crude fat	1.46 ± 0.07ab	1.52 ± 0.15ab	1.71 ± 0.04a	1.51 ± 0.07ab	1.43 ± 0.04ab	1.36 ± 0.02b	*F*_5,24_ = 2.378, *p* < 0.05
	Crude ash	1.80 ± 0.03bc	1.84 ± 0.04bc	2.09 ± 0.08a	1.99 ± 0.06ab	1.90 ± 0.03abc	1.69 ± 0.03c	*F*_5,24_ = 7.850, *p* < 0.05

*Note:* Data marked with different lowercase letters in the same column indicate significant differences (Tukey‐B, *p* < 0.05). Results are presented as mean ± standard error (SE).

Regarding muscle measurements, the crude protein content in the highly decomposed degreased *H. illucens* meal group (T_2_) was significantly lower than that in the control group (*p* < 0.05). Furthermore, the crude lipid and crude ash contents in the moderately decomposed (T_1_) and highly decomposed (T_2_) groups were significantly reduced compared to the control (*p* < 0.05). No significant differences were observed in whole shrimp proximate composition among all groups (*p* > 0.05) (Table 6).

**TABLE 6:** Effect of freshness of degreased *Hermetia illucens* meal on nutrient content in *Macrobrachium nipponense* (%).

Groups		CK	T_1_	T_2_	
Whole shrimp	Moisture	77.37 ± 1.55a	78.44 ± 1.39a	79.16 ± 0.95a	*F*_2,12_ = 0.467, *p* = 0.68
	Crude protein	18.69 ± 0.66a	17.82 ± 0.16a	17.35 ± 0.35a	*F*_2,12_ = 2.382, *p* = 0.135
	Crude fat	1.97 ± 0.09a	1.74 ± 0.09a	1.77 ± 0.06a	*F*_2,12_ = 2.344, *p* = 0.138
	Crude ash	1.52 ± 0.06a	1.60 ± 0.06a	1.62 ± 0.11a	*F*_2,12_ = 0.489, *p* = 0.625
Shrimp muscle	Moisture	72.64 ± 1.16a	74.85 ± 2.27a	75.89 ± 2.12a	*F*_2,12_ = 0.751, *p* = 0.493
	Crude protein	23.47 ± 0.69a	21.91 ± 0.42ab	21.01 ± 0.77b	*F*_2,12_ = 3.767, *p* < 0.05
	Crude fat	1.86 ± 0.11a	1.60 ± 0.07ab	1.51 ± 0.05b	*F*_2,12_ = 5.174, *p* < 0.05
	Crude ash	1.38 ± 0.04b	1.48 ± 0.05b	1.64 ± 0.03a	*F*_2,12_ = 11.952, *p* < 0.05

*Note:* Data marked with different lowercase letters in the same column indicate significant differences (Tukey‐B, *p* < 0.05). Results are presented as mean ± standard error (SE).

### 3.3 Amino Acid Content of *M. nipponense*

In *M. nipponense* , we identified nine essential amino acids, including lysine, phenylalanine, threonine, isoleucine, leucine, valine, methionine, tryptophan, and histidine, along with 10 non‐essential amino acids, including arginine, aspartic acid, alanine, glutamic acid, glycine, serine, cystine, cysteine, proline, and tyrosine (Table 7). When degreased *H. illucens* meal replaced 20% fish meal, the levels of essential amino acids lysine, phenylalanine, threonine, isoleucine, leucine, valine, methionine, and tryptophan in *M. nipponense* muscle were notably greater than those observed in the control group (*p* < 0.05). Although the concentration of histidine was elevated compared to the control group, the variation was not statistically significant (*p* > 0.05). In a similar vein, the concentrations of non‐essential amino acids such as aspartic acid, alanine, glutamic acid, cysteine, proline, and tyrosine in the abdominal muscle of *M. nipponense* shrimp were markedly higher than those in the control group when 20% of the fish meal was substituted with degreased *H. illucens* meal (*p* < 0.05). The total amino acid and total essential amino acid contents in the T_20_ group were significantly higher than those in all other groups (*p* < 0.05).

**TABLE 7:** Effect of the substitution ratio of degreased *Hermetia illucens* meal with fish meal on the contents of amino acids in *Macrobrachium nipponense* muscle (mg/kg).

Amino acids		CK	T_10_	T_20_	T_30_	T_40_	T_50_	
Essential amino acids	Lys	16.73 ± 0.65b	18.03 ± 0.52ab	19.67 ± 0.83a	17.59 ± 0.30ab	17.31 ± 0.68ab	17.20 ± 0.69ab	*F*_5,24_ = 2.709, *p* < 0.05
	Phe	7.55 ± 0.06ab	7.16 ± 0.54b	8.69 ± 0.64a	7.71 ± 0.43ab	7.08 ± 0.25b	6.73 ± 0.52b	*F*_5,24_ = 2.273, *p* < 0.05
	Thr	6.29 ± 0.09b	6.52 ± 0.39b	8.37 ± 0.25a	6.19 ± 0.46b	6.88 ± 0.24b	6.05 ± 0.41b	*F*_5,24_ = 6.746, *p* < 0.05
	Ile	6.62 ± 0.43ab	6.61 ± 0.38ab	8.31 ± 0.66a	6.89 ± 0.41ab	6.63 ± 0.38ab	5.91 ± 0.18b	*F*_5,24_ = 3.372, *p* < 0.05
	Leu	7.09 ± 0.35b	6.90 ± 0.39b	9.19 ± 0.46a	6.90 ± 0.34b	6.60 ± 0.15b	6.57 ± 0.22b	*F*_5,24_ = 8.619, *p* < 0.05
	Val	8.29 ± 0.39ab	8.61 ± 0.57ab	10.43 ± 0.69a	8.17 ± 0.54ab	8.09 ± 0.41b	7.75 ± 0.51b	*F*_5,24_ = 3.308, *p* < 0.05
	Met	2.28 ± 0.09b	2.31 ± 0.08b	4.13 ± 0.35a	3.23 ± 0.05ab	2.61 ± 0.36b	2.49 ± 0.32b	*F*_5,24_ = 8.382, *p* < 0.05
	Trp	2.08 ± 0.33b	2.63 ± 0.24b	4.31 ± 0.42a	3.38 ± 0.25ab	3.36 ± 0.27ab	3.12 ± 0.45ab	*F*_5,24_ = 5.105, *p* < 0.05
	His	3.62 ± 0.36a	3.62 ± 0.37a	4.66 ± 0.48a	3.44 ± 0.47a	3.03 ± 0.27a	2.91 ± 0.26a	*F*_5,24_ = 2.399, *p* = 0.07
Non‐essential amino acids	Arg	15.81 ± 0.19a	15.93 ± 0.15a	16.60 ± 0.67a	15.87 ± 0.14a	15.99 ± 0.07a	15.76 ± 0.12a	*F*_5,24_ = 1.034, *p* = 0.421
	Asp	18.51 ± 0.12b	18.65 ± 0.35b	21.33 ± 0.95a	18.92 ± 0.50b	18.52 ± 0.17b	18.44 ± 0.12b	*F*_5,24_ = 5.657, *p* < 0.05
	Ala	12.13 ± 0.78ab	12.24 ± 0.29ab	13.07 ± 0.31a	11.80 ± 0.62ab	11.70 ± 0.61ab	10.63 ± 0.51b	*F*_5,24_ = 2.135, *p* < 0.05
	Glu	20.94 ± 0.90b	20.74 ± 0.62b	23.19 ± 0.13a	22.24 ± 0.71ab	20.69 ± 0.70b	20.38 ± 0.84b	*F*_5,24_ = 2.518, *p* < 0.05
	Gly	15.52 ± 0.12a	15.57 ± 0.13a	16.29 ± 0.41a	16.06 ± 0.45a	15.56 ± 0.10a	15.45 ± 0.09a	*F*_5,24_ = 1.731, *p* = 0.166
	Ser	7.42 ± 0.14a	7.25 ± 0.10a	7.25 ± 0.06a	7.22 ± 0.06a	7.25 ± 0.11a	7.24 ± 0.05a	*F*_5,24_ = 0.625, *p* = 0.682
	(Cys)_2_	1.44 ± 0.09a	1.43 ± 0.11a	1.43 ± 0.08a	1.48 ± 0.13a	1.43 ± 0.06a	1.47 ± 0.09a	*F*_5,24_ = 0.056, *p* = 0.998
	Cys	3.52 ± 0.16b	4.83 ± 0.44ab	6.25 ± 0.41a	4.80 ± 0.40ab	4.60 ± 0.46ab	4.40 ± 0.44b	*F*_5,24_ = 4.943, *p* < 0.05
	Pro	6.77 ± 0.07ab	6.95 ± 0.17ab	7.68 ± 0.51a	6.63 ± 0.12ab	6.41 ± 0.23b	6.16 ± 0.36b	*F*_5,24_ = 3.382, *p* < 0.05
	Tyr	3.10 ± 0.08b	3.57 ± 0.28ab	4.62 ± 0.58a	3.09 ± 0.10b	3.02 ± 0.06b	2.89 ± 0.05b	*F*_5,24_ = 5.775, *p* < 0.05
Total amino acids		148.07 ± 1.71b	153.40 ± 1.47b	183.79 ± 1.75a	155.85 ± 3.09b	154.54 ± 1.92b	150.69 ± 1.45b	*F*_5,24_ = 43.750, *p* < 0.05
Essential amino acids		60.54 ± 0.49b	62.40 ± 0.75b	77.77 ± 1.73a	63.51 ± 1.42b	61.61 ± 1.19b	58.65 ± 1.68b	*F*_5,24_ = 28.569, *p* < 0.05
EAA/TAA, %		40.90 ± 0.34ab	40.68 ± 0.39ab	42.30 ± 0.66a	40.74 ± 0.22ab	39.86 ± 0.45b	38.89 ± 076b	*F*_5,24_ = 5.079, *p* < 0.05
Flavor amino acids (Glu, Asp)		39.45 ± 0.93b	39.39 ± 0.48b	44.52 ± 0.95a	41.16 ± 1.01ab	39.21 ± 0.63b	38.81 ± 0.80b	*F*_5,24_ = 6.951, *p* < 0.05

*Note:* (Cys)_2_ represents cystine (the oxidized dimer) and Cys represents cysteine. The below is the same. Data marked with different lowercase letters in the same column indicate significant differences (Tukey‐B, *p* < 0.05). Results are presented as mean ± standard error (SE).

In *M. nipponense* , nine essential and 10 non‐essential amino acids were identified (Table 8). The concentrations of essential amino acids such as isoleucine, tryptophan, and histidine in the medium‐decomposed (T_1_) and highly decomposed (T_2_) degreased *H. illucens* meal treatments were notably lower compared to the control group (*p* < 0.05). Similarly, the levels of essential amino acids phenylalanine, valine, and methionine in the T_2_ treatment were significantly reduced relative to the control (*p* < 0.05). For non‐essential amino acids, arginine, alanine, cysteine, proline, and tyrosine in both T_1_ and T_2_ treatments were significantly lower than those in the control (*p* < 0.05). Furthermore, the T_2_ treatment showed significantly reduced levels of non‐essential amino acids glutamic acid and glycine compared to the control (*p* < 0.05). In the moderately (T_1_) and highly (T_2_) decomposed degreased *H. illucens* meal treatments, the concentrations of non‐essential amino acids aspartic acid, serine, and cystine were lower than those in the control, although these differences were not statistically significant (*p* > 0.05). Total amino acid and total essential amino acid contents decreased significantly with increasing degree of decomposition (*p* < 0.05).

**TABLE 8:** Effect of freshness of degreased *Hermetia illucens* meal on the contents of amino acids in *Macrobrachium nipponense* muscle (mg/kg).

Amino acids		CK	T_1_	T_2_	
Essential amino acids	Lys	16.61 ± 0.56a	16.52 ± 0.83a	15.91 ± 0.75a	*F*_2,12_ = 0.285, *p* = 0.757
	Phe	7.37 ± 0.56a	7.27 ± 0.41a	5.08 ± 0.53b	*F*_2,12_ = 6.621, *p* < 0.05
	Thr	6.91 ± 074a	5.98 ± 0.40a	4.97 ± 0.46a	*F*_2,12_ = 3.012, *p* = 0.087
	Ile	7.77 ± 0.55a	5.48 ± 0.35ab	4.83 ± 0.92b	*F*_2,12_ = 5.628, *p* < 0.05
	Leu	7.27 ± 0.61a	7.06 ± 0.50a	6.91 ± 0.50a	*F*_2,12_ = 0.113, *p* = 0.894
	Val	9.12 ± 0.43a	8.09 ± 0.36a	6.56 ± 0.53b	*F*_2,12_ = 8.327, *p* < 0.05
	Met	2.89 ± 0.36a	2.07 ± 0.39ab	1.24 ± 0.05b	*F*_2,12_ = 7.187, *p* < 0.05
	His	4.77 ± 0.37a	3.18 ± 0.28b	3.11 ± 0.16b	*F*_2,12_ = 10.790, *p* < 0.05
	Trp	4.49 ± 0.25a	3.55 ± 0.20b	2.71 ± 0.29b	*F*_2,12_ = 12.978, *p* < 0.05
Non‐essential amino acids	Arg	16.15 ± 0.60a	13.52 ± 0.34b	12.61 ± 0.41b	*F*_2,12_ = 15.905, *p* < 0.05
	Asp	19.83 ± 0.47a	19.27 ± 1.03a	18.32 ± 0.69a	*F*_2,12_ = 1.008, *p* = 0.394
	Ala	14.79 ± 0.47a	13.17 ± 0.22ab	11.65 ± 0.55c	*F*_2,12_ = 12.990, *p* < 0.05
	Glu	22.91 ± 1.36a	20.10 ± 1.23ab	18.52 ± 0.48b	*F*_2,12_ = 4.155, *p* < 0.05
	Gly	17.53 ± 0.69a	15.65 ± 0.89ab	13.82 ± 0.79b	*F*_2,12_ = 5.443, *p* < 0.05
	Ser	7.98 ± 0.83a	7.50 ± 0.64a	7.39 ± 0.74a	*F*_2,12_ = 0.183, *p* = 0.835
	(Cys)_2_	1.59 ± 0.24a	1.58 ± 0.19a	1.55 ± 0.14a	*F*_2,12_ = 0.012, *p* = 0.988
	Cys	3.56 ± 0.20a	2.87 ± 0.19a	1.77 ± 0.27b	*F*_2,12_ = 16.473, *p* < 0.05
	Pro	7.45 ± 0.51a	5.72 ± 0.45b	5.25 ± 0.24b	*F*_2,12_ = 7.685, *p* < 0.05
	Tyr	6.55 ± 0.37a	5.41 ± 0.33ab	4.33 ± 0.37b	*F*_2,12_ = 9.501, *p* < 0.05
Total amino acids		185.55 ± 0.97a	164.00 ± 2.80b	146.55 ± 3.14c	*F*_2,12_ = 61.486, *p* < 0.05
Essential amino acids		67.21 ± 0.98a	59.21 ± 1.54b	51.34 ± 1.82c	*F*_2,12_ = 28.380, *p* < 0.05
EAA/TAA, %		51.60 ± 0.81a	50.73 ± 1.04a	49.04 ± 0.82a	*F*_2,12_ = 2.117, *p* = 0.163
Flavor amino acids (Glu, Asp)		42.74 ± 1.66a	39.37 ± 2.13a	36.83 ± 0.91a	*F*_2,12_ = 3.248, *p* = 0.075

*Note:* Data marked with different lowercase letters in the same column indicate significant differences (Tukey‐B, *p* < 0.05). Results are presented as mean ± standard error (SE).

### 3.4 Fatty Acid Content of *M. nipponense*

In *M. nipponense* , seven saturated fatty acids, five monounsaturated fatty acids, and six polyunsaturated fatty acids were identified (Table 9). Replacing 20% of fish meal with degreased *H. illucens* meal led to significantly increased levels of saturated fatty acids such as myristic acid (C14:0), pentadecanoic acid (C15:0), and margaric acid (C17:0) in the prawn's abdominal muscle compared to the control group (*p* < 0.05). Likewise, the monounsaturated fatty acids palmitoleic acid (C16:1) and heptadecenoic acid (C17:1) were notably higher than in the control (*p* < 0.05), although no significant differences were observed between T_20_ and the control (*p* > 0.05). Additionally, polyunsaturated fatty acids such as linoleic acid (C18:2n‐6), α‐linolenic acid (C18:3n‐3), docosapentaenoic acid (C22:5n‐3), and docosahexaenoic acid (C22:6n‐3) were significantly elevated when 20% of fish meal was substituted with degreased *H. illucens* meal (*p* < 0.05). Total polyunsaturated fatty acid content in the T_20_ group was significantly higher than that in all other groups (*p* < 0.05).

**TABLE 9:** Effect of the substitution ratio of degreased *Hermetia illucens* meal on the contents of fatty acids in *Macrobrachium nipponense* muscle (mg/kg).

Fatty acids	CK	T_10_	T_20_	T_30_	T_40_	T_50_	
C14:0	5.48 ± 0.22ab	5.54 ± 0.29ab	7.27 ± 0.79a	5.47 ± 0.29ab	5.24 ± 0.44b	4.85 ± 0.40b	*F*_5,24_ = 3.531, *p* < 0.05
C15:0	5.31 ± 0.33ab	5.61 ± 0.22ab	6.66 ± 0.40a	5.82 ± 0.31ab	4.79 ± 0.05b	4.91 ± 0.49b	*F*_5,24_ = 4.339, *p* < 0.05
C16:0	17.62 ± 0.20a	17.61 ± 0.15a	17.60 ± 0.28a	17.40 ± 0.31a	17.35 ± 0.30a	17.35 ± 0.30a	*F*_5,24_ = 0.265, *p* = 0.928
C17:0	0.53 ± 0.02b	0.58 ± 0.05b	0.80 ± 0.08a	0.60 ± 0.02b	0.55 ± 0.03b	0.51 ± 0.04b	*F*_5,24_ = 5.689, *p* < 0.05
C18:0	4.47 ± 0.21a	4.49 ± 0.12a	4.63 ± 0.13a	4.55 ± 0.15a	4.44 ± 0.11a	4.42 ± 0.11a	*F*_5,24_ = 0.298, *p* = 0.909
C20:0	0.46 ± 0.04a	0.47 ± 0.06a	0.54 ± 0.05a	0.50 ± 0.05a	0.50 ± 0.04a	0.45 ± 0.03a	*F*_5,24_ = 0.520, *p* = 0.758
C22:0	4.96 ± 0.22a	4.96 ± 0.26a	4.97 ± 0.16a	4.92 ± 0.08a	4.89 ± 0.08a	4.93 ± 0.22a	*F*_5,24_ = 0.035, *p* = 0.999
C14:1	0.49 ± 0.02a	0.49 ± 0.03a	0.50 ± 0.02a	0.49 ± 0.02a	0.49 ± 0.02a	0.48 ± 0.03a	*F*_5,24_ = 0.134, *p* = 0.983
C16:1	2.49 ± 0.10b	2.89 ± 0.39ab	3.97 ± 1.03a	2.94 ± 0.55ab	2.46 ± 0.20b	2.49 ± 0.09b	*F*_5,24_ = 4.443, *p* < 0.05
C17:1	0.44 ± 0.04ab	0.52 ± 0.04ab	0.62 ± 0.02a	0.50 ± 0.05ab	0.46 ± 0.05ab	0.36 ± 0.07b	*F*_5,24_ = 2.900, *p* < 0.05
C18:1	13.81 ± 1.07a	14.41 ± 1.19a	16.83 ± 0.21a	15.05 ± 0.75a	13.77 ± 1.29a	13.65 ± 1.01a	*F*_5,24_ = 1.528, *p* = 0.218
C20:1	0.41 ± 0.03a	0.40 ± 0.03a	0.49 ± 0.04a	0.42 ± 0.02a	0.41 ± 0.02a	0.40 ± 0.02a	*F*_5,24_ = 1.400, *p* = 0.260
C18:2n‐6	4.23 ± 0.40b	4.64 ± 0.43ab	6.35 ± 0.38a	5.07 ± 0.37ab	4.35 ± 0.44b	4.15 ± 0.51b	*F*_5,24_ = 3.820, *p* < 0.05
C18:3n‐3	5.70 ± 0.62b	6.71 ± 0.38ab	8.25 ± 0.58a	6.74 ± 0.45ab	6.24 ± 0.67ab	5.66 ± 0.62b	*F*_5,24_ = 2.864, *p* < 0.05
C20:4n‐6	0.51 ± 0.03a	0.52 ± 0.02a	0.54 ± 0.02a	0.51 ± 0.03a	0.53 ± 0.03a	0.49 ± 0.02a	*F*_5,24_ = 0.448, *p* = 0.810
C20:5n‐3	6.87 ± 0.47a	6.88 ± 0.41a	8.41 ± 0.72a	7.31 ± 0.11a	7.16 ± 0.15a	7.02 ± 0.13a	*F*_5,24_ = 2.157, *p* = 0.093
C22:5n‐3	1.38 ± 0.08b	1.43 ± 0.05b	1.71 ± 0.05a	1.48 ± 0.06ab	1.39 ± 0.05b	1.34 ± 0.05b	*F*_5,24_ = 5.552, *p* < 0.05
C22:6n‐3	21.16 ± 0.61b	23.34 ± 0.39ab	25.52 ± 0.76a	23.22 ± 0.99ab	22.98 ± 1.14ab	21.54 ± 0.51b	*F*_5,24_ = 3.993, *p* < 0.05
∑PUFA	39.84 ± 1.74b	43.52 ± 0.64b	50.78 ± 1.50a	44.33 ± 1.33b	42.65 ± 1.50b	40.20 ± 1.04b	*F*_5,24_ = 8.748, *p* < 0.05

*Note:* Data marked with different lowercase letters in the same column indicate significant differences (Tukey‐B, *p* < 0.05). Results are presented as mean ± standard error (SE).

The types of saturated, monounsaturated, and polyunsaturated fatty acids in *M. nipponense* were seven, five, and six, respectively (Table 10). The content of docosanoic acid (C22:0) in the moderate (T_1_) and high (T_2_) decomposition treatments was significantly reduced compared to the control (*p* < 0.05). The pentadecanoic acid (C15:0) level in the highly decomposed degreased *H. illucens* meal treatment (T_2_) was also significantly lower than in the control (*p* < 0.05). Although the levels of myristic, palmitic, margaric, stearic, and arachidic acids in the moderately (T_1_) and highly (T_2_) decomposed treatments were lower than in the control, these differences were not significant (*p* > 0.05). The oleic acid (C18:1) content in the moderate (T_1_) and high (T_2_) decomposed treatments was significantly less than in the control (*p* < 0.05). The levels of monounsaturated fatty acids such as myristoleic acid, palmitoleic acid, heptadecenoic acid, and gadoleic acid were lower than those in the control group, but these differences were not significant (*p* > 0.05). The polyunsaturated fatty acids linoleic acid (C18:2n‐6), eicosapentaenoic acid (C20:5n‐3), and docosahexaenoic acid (C22:6n‐3) in the moderate (T_1_) and high (T_2_) decomposed treatments were significantly lower than in the control (*p* < 0.05). The levels of α‐linolenic acid (C18:3n‐3) in the highly decomposed treatment (T_2_) were significantly reduced compared to the control (*p* < 0.05). The arachidonic acid (C20:4n‐6) and docosapentaenoic acid (C22:5n‐3) content in the moderate (T_1_) and high (T_2_) decomposed treatments was lower than in the control, but the difference was not significant (*p* > 0.05). Total polyunsaturated fatty acid content in T_1_ and T_2_ was significantly lower than that in the control (*p* < 0.05).

**TABLE 10:** Effect of freshness of degreased *Hermetia illucens* meal on the contents of fatty acids in *Macrobrachium nipponense* muscle (mg/kg).

Fatty acids	CK	T_1_	T_2_	
C14:0	5.91 ± 0.67a	5.35 ± 0.23a	4.84 ± 0.32a	*F*_2,12_ = 1.409, *p* = 0.282
C15:0	6.33 ± 0.60a	5.27 ± 0.44ab	4.04 ± 0.23b	*F*_2,12_ = 6.498, *p* < 0.05
C16:0	16.03 ± 0.79a	15.72 ± 1.11a	15.19 ± 0.90a	*F*_2,12_ = 0.204, *p* = 0.818
C17:0	0.49 ± 0.04a	0.46 ± 0.05a	0.42 ± 0.03a	*F*_2,12_ = 0.698, *p* = 0.517
C18:0	4.90 ± 0.48a	3.96 ± 0.23a	3.84 ± 0.24a	*F*_2,12_ = 2.942, *p* = 0.091
C20:0	0.52 ± 0.05a	0.49 ± 0.08a	0.49 ± 0.05a	*F*_2,12_ = 0.123, *p* = 0.885
C22:0	6.24 ± 0.39a	4.40 ± 0.31b	4.23 ± 0.38b	*F*_2,12_ = 9.503, *p* < 0.05
C14:1	0.53 ± 0.05a	0.49 ± 0.06a	0.48 ± 0.05a	*F*_2,12_ = 0.251, *p* = 0.782
C16:1	2.95 ± 0.24a	2.93 ± 0.13a	2.78 ± 0.13a	*F*_2,12_ = 0.266, *p* = 0.771
C17:1	0.42 ± 0.04a	0.40 ± 0.05a	0.38 ± 0.04a	*F*_2,12_ = 0.249, *p* = 0.783
C18:1	15.82 ± 0.54a	12.91 ± 0.66b	12.77 ± 0.67b	*F*_2,12_ = 7.462, *p* < 0.05
C20:1	0.39 ± 0.08a	0.35 ± 0.03a	0.31 ± 0.02a	*F*_2,12_ = 0.638, *p* = 0.545
C18:2n‐6	5.42 ± 0.17a	4.55 ± 0.27b	3.63 ± 0.11c	*F*_2,12_ = 20.916, *p* < 0.05
C18:3n‐3	7.10 ± 0.42a	6.25 ± 0.28ab	5.63 ± 0.24b	*F*_2,12_ = 5.238, *p* < 0.05
C20:4n‐6	0.52 ± 0.04a	0.52 ± 0.06a	0.40 ± 0.06b	*F*_2,12_ = 1.468, *p* = 0.269
C20:5n‐3	7.21 ± 0.30a	6.00 ± 0.23b	5.72 ± 0.26b	*F*_2,12_ = 8.913, *p* < 0.05
C22:5n‐3	1.62 ± 0.13a	1.46 ± 0.10a	1.45 ± 0.12a	*F*_2,12_ = 0.703, *p* = 0.514
C22:6n‐3	23.79 ± 0.67a	21.33 ± 0.46b	20.59 ± 0.69b	*F*_2,12_ = 7.350, *p* < 0.05
∑PUFA	45.65 ± 1.01a	40.09 ± 0.51b	37.42 ± 0.92b	*F*_2,12_ = 25.720, *p* < 0.05

*Note:* Data marked with different lowercase letters in the same column indicate significant differences (Tukey‐B, *p* < 0.05). Results are presented as mean ± standard error (SE).

### 3.5 Mineral Element Content of *M. nipponense*

The mineral elements in *M. nipponense* include sodium, potassium, magnesium, calcium, manganese, iron, zinc, selenium, and copper (Table 11). When 20% of fishmeal was replaced with degreased *H. illucens* meal, the potassium, calcium, and iron contents in *M. nipponense* abdominal muscle showed a significant increase compared to the control group (*p* < 0.05). Although the levels of sodium, magnesium, zinc, selenium, and manganese in the abdominal muscle of *M. nipponense* were elevated compared to the control, these differences were not statistically significant (*p* > 0.05). Replacing 40% and 50% of fishmeal with degreased *H. illucens* meal resulted in a copper content in *M. nipponense* abdominal muscle that was significantly greater than that of the control group (*p* < 0.05).

**TABLE 11:** Effect of the substitution ratio of degreased *Hermetia illucens* meal on the contents of mineral elements in *Macrobrachium nipponense* muscle (mg/kg).

Metallic element	CK	T_10_	T_20_	T_30_	T_40_	T_50_	
Na	25.78 ± 0.57a	25.69 ± 0.60a	25.84 ± 0.61a	25.57 ± 0.51a	25.49 ± 0.54a	25.47 ± 0.70a	*F*_5,24_ = 0.067, *p* = 0.997
K	1716.57 ± 17.94b	1815.13 ± 45.27b	2045.27 ± 78.24a	1832.47 ± 46.92b	1747.47 ± 33.92b	1472.38 ± 39.77b	*F*_5,24_ = 6.510, *p* < 0.05
Mg	163.54 ± 3.71a	168.85 ± 8.63a	174.94 ± 7.44a	164.50 ± 4.35a	164.11 ± 3.77a	166.46 ± 7.73a	*F*_5,24_ = 0.473, *p* = 0.792
Ca	756.08 ± 11.94b	759.05 ± 10.21b	827.13 ± 20.34a	777.02 ± 18.51ab	763.74 ± 14.07ab	762.34 ± 14.30ab	*F*_5,24_ = 3.088, *p* < 0.05
Mn	0.48 ± 0.04a	0.44 ± 0.05a	0.46 ± 0.04a	0.44 ± 0.07a	0.42 ± 0.05a	0.43 ± 0.04a	*F*_5,24_ = 0.195, *p* = 0.961
Fe	3.61 ± 0.13b	4.07 ± 0.18b	4.77 ± 0.35a	3.90 ± 0.17b	3.83 ± 0.10b	3.57 ± 0.10b	*F*_5,24_ = 5.186, *p* < 0.05
Zn	51.59 ± 3.42a	54.46 ± 2.55a	59.09 ± 2.02a	53.25 ± 2.10a	51.50 ± 2.44a	50.60 ± 3.26a	*F*_5,24_ = 1.369, *p* = 0.271
Se	0.72 ± 0.06a	0.78 ± 0.04a	0.87 ± 0.11a	0.82 ± 0.04a	0.72 ± 0.06a	0.66 ± 0.04a	*F*_5,24_ = 1.392, *p* = 0.263
Cu	0.24 ± 0.02c	0.24 ± 0.04c	0.27 ± 0.04bc	0.36 ± 0.07abc	0.46 ± 0.04ab	0.50 ± 0.04a	*F*_5,24_ = 6.305, *p* < 0.05

*Note:* Data marked with different lowercase letters in the same column indicate significant differences (Tukey‐B, *p* < 0.05). Results are presented as mean ± standard error (SE).

The mineral elements in *M. nipponense* include sodium, potassium, magnesium, calcium, manganese, iron, zinc, selenium, and copper (Table 12). In the shrimp muscle from the moderately (T_1_) and highly (T_2_) decomposed degreased *H. illucens* meal treatments, the magnesium and calcium contents were significantly higher than those in the control (*p* < 0.05). In the highly decomposed degreased *H. illucens* meal treatment (T_2_), the potassium and zinc contents in the shrimp muscle were notably greater compared to the control group (*p* < 0.05). In the treatments involving moderately (T_1_) and highly (T_2_) decomposed degreased *H. illucens* meal, the copper concentration in shrimp muscle was markedly elevated relative to the control (*p* < 0.05). No significant differences were observed in sodium, manganese, iron, or selenium contents among all groups (*p* > 0.05).

**TABLE 12:** Effect of freshness of degreased *Hermetia illucens* meal on the contents of mineral elements in *Macrobrachium nipponense* muscle (mg/kg).

Metallic element	CK	T_1_	T_2_	
Na	27.01 ± 1.39a	29.42 ± 1.58a	31.41 ± 1.90a	*F*_2,12_ = 1.810, *p* = 0.206
K	1644.77 ± 36.84b	1760.11 ± 57.11ab	1864.59 ± 38.36a	*F*_2,12_ = 5.956, *p* < 0.05
Mg	139.56 ± 4.25b	166.23 ± 2.25a	177.65 ± 2.09a	*F*_2,12_ = 41.627, *p* < 0.05
Ca	646.47 ± 10.88c	718.21 ± 18.20b	814.30 ± 22.18a	*F*_2,12_ = 22.593, *p* < 0.05
Mn	0.50 ± 0.05a	0.48 ± 0.04a	0.51 ± 0.04a	*F*_2,12_ = 0.076, *p* = 0.928
Fe	3.49 ± 0.33a	3.47 ± 0.25a	3.72 ± 0.38a	*F*_2,12_ = 0.185, *p* = 0.833
Zn	58.09 ± 2.09b	63.59 ± 4.47ab	72.98 ± 4.31a	*F*_2,12_ = 3.963, *p* < 0.05
Se	0.58 ± 0.05a	0.56 ± 0.03a	0.60 ± 0.07a	*F*_2,12_ = 0.173, *p* = 0.843
Cu	0.36 ± 0.13b	0.52 ± 0.10b	0.83 ± 0.03a	*F*_2,12_ = 28.025, *p* < 0.05

*Note:* Data marked with different lowercase letters in the same column indicate significant differences (Tukey‐B, *p* < 0.05). Results are presented as mean ± standard error (SE).

### 3.6 Amino Acid Transport and Antioxidant Capacity in Serum and Hepatopancreas

The substitution of fishmeal with degreased *H. illucens* meal had a notable impact on the amino acid transport and antioxidant levels in the serum and hepatopancreas of *M. nipponense* (Table 13). When 20% and 30% of fishmeal were replaced with degreased *H. illucens* meal, the superoxide dismutase (SOD) activity in the hepatopancreas was significantly elevated compared to the control group (*p* < 0.05). The aspartate aminotransferase (AST) activity in the hepatopancreas was significantly higher in the T_50_ group than in the control (*p* < 0.05). No significant differences were observed in hepatopancreatic alanine aminotransferase (ALT), malondialdehyde (MDA), or glutathione peroxidase (GSH‐Px) activities among all groups (*p* > 0.05). At a 20% replacement level, the hemolymph showed significantly increased activities of acid phosphatase (ACP) and lysozyme (LZM), compared to the control (*p* < 0.05). Alkaline phosphatase (ALP) activity in the hemolymph exceeded that of the control at replacement levels of 10%, 20%, 30%, 40%, and 50%, although these differences were not statistically significant (*p* > 0.05).

**TABLE 13:** Effects of the substitution ratio of degreased *Hermetia illucens* meal on the antioxidant capacity in *Macrobrachium nipponense* .

Group	Serum biochemical parameters	CK	T_10_	T_20_	T_30_	T_40_	T_50_	
Hepatopancreas	Aspartate aminotransferase (AST)/(U/g prot)	3.57 ± 0.06b	3.56 ± 0.06b	3.55 ± 0.06b	3.95 ± 0.21ab	3.95 ± 0.11ab	4.16 ± 0.25a	*F*_5,24_ = 3.164, *p* < 0.05
	Alanine aminotransferase (ALT)/(U/g prot)	5.25 ± 0.13a	5.24 ± 0.15a	5.26 ± 0.22a	5.46 ± 0.35a	5.72 ± 0.33a	5.91 ± 0.28a	*F*_5,24_ = 1.235, *p* = 0.324
	Malondialdehyde (MDA)/(nmol/mg prot)	5.67 ± 0.15a	5.70 ± 0.16a	5.65 ± 0.20a	5.74 ± 0.26a	6.20 ± 0.36a	6.24 ± 0.41a	*F*_5,24_ = 1.009, *p* = 0.434
	Superoxide dismutase (SOD)/(U/mg prot)	22.20 ± 1.41c	23.64 ± 0.86abc	26.69 ± 1.02a	25.93 ± 1.04ab	23.52 ± 0.95abc	22.81 ± 1.04bc	*F*_5,24_ = 2.785, *p* < 0.05
	Glutathione peroxidase (GSH‐PX)/(U/mg prot)	158.41 ± 5.00a	160.79 ± 4.57a	179.46 ± 4.85a	166.91 ± 5.54a	165.68 ± 5.29a	164.05 ± 5.96a	*F*_5,24_ = 1.984, *p* = 0.118
Hemolymph	Aspartate aminotransferase (AST)/(U/g prot)	19.44 ± 0.75a	19.38 ± 1.52a	20.07 ± 0.87a	20.70 ± 1.07a	20.74 ± 1.08a	21.14 ± 1.01a	*F*_5,24_ = 0.461, *p* = 0.801
	Alanine aminotransferase (ALT)/(U/g prot)	22.00 ± 1.52a	21.92 ± 0.89a	22.50 ± 0.86a	23.75 ± 0.93a	23.82 ± 0.71a	23.86 ± 0.88a	*F*_5,24_ = 0.881, *p* = 0.509
	Acid phosphatase (ACP)/(U/L)	8.15 ± 0.38b	8.77 ± 0.45ab	10.48 ± 0.71a	9.75 ± 0.26ab	9.75 ± 0.33ab	8.94 ± 0.30ab	*F*_5,24_ = 3.791, *p* < 0.05
	Alkaline phosphatase (ALP)/(U/L)	12.81 ± 0.55a	13.01 ± 0.76a	13.06 ± 0.45a	13.15 ± 0.48a	13.89 ± 1.07a	13.75 ± 0.49a	*F*_5,24_ = 0.422, *p* = 0.829
	Lysozyme (LZM)/(U/mg prot)	2.59 ± 0.12b	2.87 ± 0.16b	4.30 ± 0.52a	2.83 ± 0.10b	2.25 ± 0.10b	2.11 ± 0.14b	*F*_5,24_ = 10.658, *p* < 0.05

*Note:* Data marked with different lowercase letters in the same column indicate significant differences (Tukey‐B, *p* < 0.05). Results are presented as mean ± standard error (SE).

The freshness of degreased *H. illucens* meal also significantly influenced amino acid transport and antioxidant capacity in the serum and hepatopancreas of *M. nipponense* (Table 14). In the treatment with moderately decomposed degreased *H. illucens* meal (T_1_), the hepatopancreas exhibited significantly higher activities of alanine aminotransferase (ALT) and superoxide dismutase (SOD), along with increased malondialdehyde (MDA) content compared to the control (*p* < 0.05). In the group receiving highly decomposed degreased *H. illucens* meal (T_2_), the activities of aspartate aminotransferase (AST) and alanine aminotransferase (ALT), as well as the MDA content in the hepatopancreas, were significantly elevated compared to the control (*p* < 0.05). In the hemolymph of the group treated with moderately decomposed degreased *H. illucens* meal (T_1_), the activities of alanine aminotransferase (ALT) and alkaline phosphatase (ALP) were significantly higher than those in the control group (*p* < 0.05). For the highly decomposed degreased *H. illucens* meal treatment (T_2_), the hemolymph showed significantly increased activities of aspartate aminotransferase (AST), alanine aminotransferase (ALT), acid phosphatase (ACP), lysozyme (LZM), and alkaline phosphatase (ALP) compared to the control (*p* < 0.05).

**TABLE 14:** Effects of freshness of degreased *Hermetia illucens* meal on antioxidant capacity in *Macrobrachium nipponense* .

Group	Serum biochemical parameters	CK	T_1_	T_2_	
Hepatopancreas	Aspartate aminotransferase (AST)/(U/g prot)	3.63 ± 0.12b	3.82 ± 0.12b	5.89 ± 0.49a	*F*_2,12_ = 17.471, *p* < 0.05
	Alanine aminotransferase (ALT)/(U/g prot)	5.51 ± 0.32b	7.52 ± 0.62a	7.94 ± 0.70a	*F*_2,12_ = 5.164, *p* < 0.05
	Malondialdehyde (MDA)/(nmol/mg prot)	5.09 ± 0.27b	8.72 ± 0.56a	9.11 ± 0.90a	*F*_2,12_ = 12.347, *p* < 0.05
	Superoxide dismutase (SOD)/(U/mg prot)	22.95 ± 1.03a	25.43 ± 0.72a	18.15 ± 0.63b	*F*_2,12_ = 20.837, *p* < 0.05
	Glutathione peroxidase (GSH‐PX)/(U/mg prot)	165.48 ± 5.55a	173.79 ± 5.68a	183.87 ± 5.95a	*F*_2,12_ = 2.586, *p* = 0.116
Hemolymph	Aspartate aminotransferase (AST)/(U/g prot)	21.06 ± 1.05b	23.77 ± 1.29ab	27.01 ± 1.05a	*F*_2,12_ = 6.886, *p* < 0.05
	Alanine aminotransferase (ALT)/(U/g prot)	22.84 ± 0.70c	26.57 ± 0.33b	29.26 ± 0.87a	*F*_2,12_ = 22.799, *p* < 0.05
	Acid phosphatase (ACP)/(U/L)	7.94 ± 0.50b	9.95 ± 0.31b	13.36 ± 1.24a	*F*_2,12_ = 11.903, *p* < 0.05
	Alkaline phosphatase (ALP)/(U/L)	12.39 ± 0.63b	24.56 ± 1.36a	25.46 ± 1.11a	*F*_2,12_ = 4.755, *p* < 0.05
	Lysozyme (LZM)/(U/mg prot)	2.81 ± 0.19b	4.46 ± 0.27b	6.76 ± 0.94a	*F*_2,12_ = 11.919, *p* < 0.05

*Note:* Data marked with different lowercase letters in the same column indicate significant differences (Tukey‐B, *p* < 0.05). Results are presented as mean ± standard error (SE).

### 3.7 Hepatopancreatic Digestive Enzyme Activities

Replacing fish meal with degreased *H. illucens* meal significantly affected the digestive enzyme activities in the hepatopancreas of *M. nipponense* , as shown in Table 15. A 20% substitution of fish meal with degreased *H. illucens* meal led to a marked increase in trypsin (TPS) and lipase (LPS) activities in the hepatopancreas, compared to the control group (*p* < 0.05). The trypsin activity in the T_50_ group was significantly lower than that in the T_20_ group (*p* < 0.05). α‐amylase (α‐AMS) activity showed no significant differences among all substitution groups (*p* > 0.05).

**TABLE 15:** Effects of the substitution ratio of degreased *Hermetia illucens* meal on digestive enzyme activities of *Macrobrachium nipponense* .

Digestive enzyme	CK	T_10_	T_20_	T_30_	T_40_	T_50_	
α‐Amylas (α‐AMS)/(U/mg prot)	0.47 ± 0.03a	0.42 ± 0.02a	0.46 ± 0.03a	0.47 ± 0.03a	0.48 ± 0.03a	0.45 ± 0.04a	*F*_5,24_ = 0438, *p* = 0.818
Trypsin (TPS)/(U/mg prot)	58.04 ± 4.23b	59.45 ± 4.85ab	78.45 ± 4.95a	68.18 ± 2.88ab	59.69 ± 5.58ab	49.27 ± 4.36b	*F*_5,24_ = 4.807, *p* < 0.05
Lipase (LPS)/(U/mg prot)	22.20 ± 0.92b	23.68 ± 1.23ab	26.88 ± 0.97a	23.98 ± 1.03ab	21.96 ± 0.66b	23.21 ± 0.91ab	*F*_5,24_ = 3.331, *p* < 0.05

*Note:* Data marked with different lowercase letters in the same column indicate significant differences (Tukey‐B, *p* < 0.05). Results are presented as mean ± standard error (SE).

The freshness of the degreased *H. illucens* meal played a crucial role in influencing the digestive enzyme activities in the hepatopancreas of *M. nipponense* , as indicated in Table 16. In the treatment involving severely decomposed degreased *H. illucens* meal (T_2_), the α‐amylase (α‐AMS), trypsin (TPS), and lipase (LPS) activity in the hepatopancreas was significantly lower compared to the control group (*p* < 0.05). For the group that received moderately decomposed degreased *H. illucens* meal (T_1_), the trypsin (TPS), and lipase (LPS) activity in the hepatopancreas significantly elevated compared to the control group (*p* < 0.05), while α‐amylase (α‐AMS) activity showed no significant difference from the control (*p* > 0.05).

**TABLE 16:** Effects of freshness of degreased *Hermetia illucens* meal on digestive enzyme activities of *Macrobrachium nipponense* .

Digestive enzyme	CK	T_1_	T_2_	
α‐Amylas (α‐AMS)/(U/mg prot)	0.52 ± 0.04a	0.50 ± 0.02a	0.39 ± 0.04b	*F*_2,12_ = 3.731, *p* < 0.05
Trypsin (TPS)/(U/mg prot)	52.13 ± 1.86ab	62.76 ± 5.14a	40.58 ± 1.96b	*F*_2,12_ = 10.838, *p* < 0.05
Lipase (LPS)/(U/mg prot)	23.20 ± 1.17b	27.66 ± 0.46a	18.68 ± 1.12c	*F*_2,12_ = 21.372, *p* < 0.05

*Note:* Data marked with different lowercase letters in the same column indicate significant differences (Tukey‐B, *p* < 0.05). Results are presented as mean ± standard error (SE).

## 4 Discussion

This study demonstrated that substituting 20% of fish meal with degreased *H. illucens* meal markedly enhanced the growth performance, crude protein content, specific essential amino acids, polyunsaturated fatty acids, and mineral concentrations in *M. nipponense* . It also significantly enhanced the digestive and nonspecific immune capacities of prawns while significantly reducing the feed conversion ratio. In contrast, the growth performance, feed efficiency, digestive ability, and nonspecific immune function of *M. nipponense* were notably diminished by moderately and highly decomposed degreased *H. illucens* meal. Additionally, these decomposed meals adversely affected nutrient accumulation in *M. nipponense* , leading to a reduction in the levels of specific monounsaturated fatty acids, polyunsaturated fatty acids, essential amino acids, and mineral elements within the muscle.

Current research indicates that using insect protein to replace fish meal protein in aquaculture can effectively improve production efficiency. Incorporating 20%–30% degreased *Periplaneta americana* meal into the diet of *O. mykiss* significantly accelerated their growth rate, with the specific growth rate and weight gain rate being significantly greater than those observed in the control group (Long et al. 2022). Juvenile *O. mykiss* exhibited significantly improved survival and growth rates when 30% of their diet consisted of degreased *P. americana* meal, compared to the control group fed with whole fish meal (Chen, Xu, et al. 2023). Additionally, with a 14% dietary inclusion of degreased *Tenebrio molitor* meal, the weight gain and specific growth rates of juvenile *O. mykiss* were markedly higher than those in the whole fish meal control group. Moreover, the efficiency of dietary protein use in *O. mykiss* surpassed that of the control group (Jeong et al. 2020). Nevertheless, incorporating 0.15%, 0.20%, and 0.25% of aqueous *P. americana* extract into the diet of *Oreochromis niloticus* did not lead to a notable enhancement in growth performance. This could be attributed to the destruction or incomplete extraction of essential nutritional components during the aqueous extraction process (Yu et al. 2023). Similarly, the inclusion of *Musca domestica* maggot meal in the diet did not result in significant changes in the growth performance metrics of *O. niloticus* when compared to the control group (Shi et al. 2015). According to research conducted by Zhang, Liu, et al. (2024), feeding degreased *H. illucens* meal to *M. rosenbergii* did not significantly impact growth and survival rates, although it did notably decrease the feed conversion ratio, thereby reducing farming expenses. The current study observed that at a 20% substitution level, the growth performance *of M. nipponense* was markedly superior to that of the control group fed with whole fish meal, and the feed conversion ratio was significantly improved compared to the control. This may be because after adding degreased *H. illucens* meal, the dietary amino acids in the feed become more balanced, which helps improve the feed palatability and enhance the digestibility of the feed by *M. nipponense* . In addition, degreased *H. illucens* meal is rich in chitosan and various antimicrobial peptides (Li, Liu, et al. 2023; Jiang et al. 2026), which may help enhance the immunity of *M. nipponense* , thereby strengthening its resistance to disease and environmental stress (Liu, Qian, et al. 2025). However, it should be noted that the T_30_, T_40_, and T_50_ groups did not exhibit significant growth improvements over the control, and the T_50_ group even showed significantly lower performance in multiple indicators compared to T_20_, suggesting that excessive replacement may introduce anti‐nutritional factors or amino acid imbalances that offset the benefits.

Aquatic feed proteins are vulnerable to environmental conditions like temperature and humidity, which can elevate the TVB‐N levels due to protein degradation, thereby diminishing the quality and taste of aquatic feed. According to Ricque‐Marie et al. (1998), the feed conversion ratio and growth performance metrics of *L. vannamei* consuming diets with fresh fish meal were notably higher compared to those of groups given moderately and highly decomposed fish meals. Hu et al. (2013) demonstrated that when diets included high‐quality fish meal (TVB‐N 100 mg/100 g), specific growth performance indicators such as weight gain rate and specific growth rate of *Lateolabrax japonicus* were markedly better than those fed with medium‐quality fish meal (TVB‐N 130 mg/100 g). Wu et al. (2025) found that the growth performance parameters of *M. rosenbergii* fed diets containing fresh fish meal or chicken meal were superior to those fed diets containing moderately and highly decomposed fish meal (TVB‐N 65, 80 mg/100 g) and chicken meal (TVB‐N 60, 68 mg/100 g), which showed that the freshness of shrimp meal can significantly affect the body weight, weight gain rate, and specific growth rate of *M. rosenbergii* . The T_1_ group (TVB‐N 65 mg/100 g) exhibited significantly lower specific growth rate (SGR) and higher feed conversion ratio (FCR) than the control, while the T_2_ group (TVB‐N 80 mg/100 g) showed even more pronounced impairments, including reduced condition factor and survival rate. Therefore, feeding diets containing low‐quality insect meal protein may reduce the profitability of *M. nipponense* farming.

The nutritional composition of aquatic products reflects the status of dietary nutrient absorption and intake and serves as the primary basis for evaluating the suitability of aquatic feeds. Currently, the nutritional quality of farmed shrimp is mainly measured by nutrient content, with a higher crude protein content often considered indicative of good meat quality (Liu et al. 2010; Ma et al. 2018). Existing studies have found that replacing fish meal with insect protein may have similar effects on different aquatic animals (Zhang 2024; Zhang, Zhou, et al. 2024). Chen, Zheng, et al. (2023) indicated that replacing fish meal with degreased *H. illucens* meal significantly reduced crude fat accumulation in the muscles of *L. vannamei* , and similar negative conclusions were reached in studies on *Penaeus monodon* and *Eriocheir sinensis* (Vasagam et al. 2005; Wu et al. 2010). A key factor contributing to this negative effect might be the substantial differences in amino acid profiles and content between insect and fish meals. However, as the inclusion level of degreased *P. americana* meal increased, the crude protein content in juvenile *O. mykiss* showed an upward trend (Long et al. 2022). We found that when 20% of the fish meal was replaced with degreased *H. illucens* meal, the crude protein and crude ash content in the abdominal muscle of *M. nipponense* were significantly higher than those in the control. This may be because degreased *H. illucens* meal optimizes the diversity of dietary protein and amino acid balance, facilitating dietary nutrient absorption, utilization, and nutrient accumulation in *M. nipponense* . However, at higher substitution levels (T_40_, T_50_), the crude protein and crude lipid contents tended to decline, suggesting a threshold effect beyond which nutrient deposition is compromised.

Dietary protein sources are crucial factors determining aquatic feed quality, and the amino acid composition and content of aquatic products can effectively reflect dietary protein quality (Zhang 2024). Increasing the inclusion of degreased *P. americana* meal significantly elevated the amino acid content in the muscles of juvenile *O. mykiss* (Long et al. 2022). When the replacement ratio of degreased *T. molitor* meal reached 45%, it had a significant negative impact on the content of essential and flavor amino acids in the muscle of *L. vannamei* (Liang et al. 2026). When degreased cricket meal was used at multiple graded levels in *P. fulvidraco* culture, the growth performance and muscle amino acid content of juveniles were unaffected by the insect meal replacement ratio (Liu et al. 2023). Zhang (2024) showed that as the proportion of degreased *P. americana* meal in the diet gradually increased, the amino acid content in the muscle of *M. rosenbergii* first increased and then decreased. The current research demonstrated that substituting fish meal with degreased *H. illucens* meal successfully enhanced the amino acid levels in the muscles of *M. nipponense* . When 20% of the fish meal was replaced, both essential and non‐essential amino acids were notably higher compared to the control group. Nevertheless, at a 50% replacement level, the amino acid content in *M. nipponense* did not differ significantly from the control, which might be attributed to alterations in dietary amino acid composition resulting from the inclusion of degreased *H. illucens* meal. The types and content of muscle fatty acids are also important factors affecting muscle taste and flavor, and diet can significantly influence fatty acid accumulation in aquatic animals. Renna et al. (2017) found that adding degreased *H. illucens* meal to the diet promoted muscle fatty acid deposition in *O. mykiss* . Fischer et al. (2022) showed that substituting fish meal with degreased *H. illucens* meal led to a notable rise in both the amount and proportion of saturated fatty acids in the muscle of *M. salmoides* . Additionally, Weng et al. (2024) found that incorporating degreased *H. illucens* meal into the diet significantly boosted the levels and composition of polyunsaturated fatty acids in *M. salmoides* muscle. In the current study, it was observed that replacing 20% of fish meal with degreased *H. illucens* meal markedly increased the levels of specific saturated fatty acids and polyunsaturated fatty acid in the muscle of *M. nipponense* . This suggests that it is possible to adjust the fatty acid composition and content of aquatic products by altering the dietary protein source.

Mineral elements are crucial for maintaining cellular physiological activities and metabolism in organisms. In this study, it was observed that substituting fish meal with degreased *H. illucens* meal at levels between 10% and 50% resulted in *M. nipponense* having Ca, Fe, and Zn contents of 759.08–827.13, 3.57–4.07, and 50.60–59.09 mg/kg, respectively. These values were lower than those documented by Zheng et al. (2022). Moreover, the presence of mineral elements in aquatic products influences the muscle's flavor characteristics; typically, potassium, sodium, calcium, and iron are associated with a salty taste, zinc with a sweet taste, and manganese and copper with a bitter taste (Ma et al. 2014; Cheng et al. 2016). In our study, the group with a 20% replacement of fish meal with degreased *H. illucens* meal showed significantly higher levels of potassium, calcium, and iron compared to the control group, while copper and manganese levels did not differ significantly between the control and the 20% replacement group, indicating that replacing fish meal with degreased *H. illucens* meal may influence the flavor and nutrition of *M. nipponense* , but does not increase bitterness at the optimal substitution level. In subsequent large‐scale farming, further optimization of the feed formulation is needed to increase the zinc content in *M. nipponense* meat by enhancing the zinc content in the feed, thereby improving the flavor of prawn products.

Dietary nutrient composition is a decisive factor affecting the nutritional composition of aquatic animals. The ash and mineral element content of aquatic animals can indirectly reflect the quality of dietary protein sources (Zhou et al. 2017; Cai et al. 2022; Li, Zeng, et al. 2023). Early research has found that low‐quality fish meal significantly increases the ash content in *G. morhua* fillets (Albrektsen et al. 2006). The present study found that moderately and highly decomposed degreased *H. illucens* meal significantly reduced the crude fat and protein content and significantly increased the crude ash content in *M. nipponense* . The current research also revealed that the use of highly decomposed degreased *H. illucens* meal led to a notable decrease in the levels of six essential amino acids (isoleucine, tryptophan, histidine, phenylalanine, valine, and methionine), seven non‐essential amino acids (arginine, alanine, cysteine, proline, tyrosine, glutamic acid, and glycine), two saturated fatty acids (pentadecanoic acid and docosanoic acid), one monounsaturated fatty acid (C18:1), and five polyunsaturated fatty acids (C18:2n‐6, C18:3n‐3, C20:4n‐6, C20:5n‐3, and C22:6n‐3) in *M. nipponense* . Conversely, it caused a significant rise in the levels of six mineral elements, namely potassium, magnesium, calcium, zinc, and copper. Therefore, a decrease in shrimp meal freshness might affect prawn nutrient content.

*Macrobrachium nipponense* primarily relies on relatively primitive humoral immune responses to resist pathogen invasion, possessing virtually no specific immune function and exhibiting an extremely primitive immune response (Zhao et al. 2025; Zhang 2024). Nutrition and immunity are two closely interacting physiological and metabolic processes in aquatic organisms. Nutritional levels directly affect the immune and disease resistance functions of aquatic organisms, and immune system function can significantly influence food intake and nutrient assimilation efficiency (Huang et al. 2012). Superoxide dismutase (SOD) and lysozyme (LZM) are important tools for shrimp defense against pathogen invasion (Xu et al. 2026). Superoxide dismutase (SOD) eliminates oxidative pressure triggered by pathogen invasion, thereby protecting the structural and functional integrity of host cells. An increase in SOD activity indicates enhanced immunity and stress resistance (Ding et al. 2013). The core function of lysozyme (LZM) is to directly destroy the cell walls of pathogens by cleaving the peptidoglycan in the cell wall, creating holes that kill pathogenic bacteria (Wang et al. 2023). Acid phosphatase (ACP) and alkaline phosphatase (ALP) enhance the phagocytic activity of immune cells, leading to the swift breakdown of invading foreign bodies and pathogens (Chen et al. 2012). Malondialdehyde (MDA) serves as a marker for lipid peroxidation and oxidative damage in organisms, with elevated levels potentially causing protein and DNA damage in arthropods and altering cell membrane integrity (Wang et al. 2023). In this study, substituting 20% of fish meal with degreased *H. illucens* meal notably boosted the activities of SOD, LZM, and ACP, while MDA levels remained unchanged. This suggests that degreased *H. illucens* meal enhances the immunity of *M. nipponense* , possibly due to the chitin and antimicrobial peptides found in *H. illucens* meal. The freshness of feed protein also significantly affects the antioxidant capacity and immune function of *M. nipponense* . In the current study, it was observed that feeding *M. nipponense* with compound diets containing moderately and highly decomposed degreased *H. illucens* meal resulted in significantly lower hepatopancreatic SOD activity in the T_2_ group compared to the fresh degreased *H. illucens* meal control group. Conversely, AST, ALT, and LZM activities in the hemolymph were significantly elevated compared to the control group. The marked increase in ACP, ALP, and MDA content in the hemolymph of *M. nipponense* highlights that the spoilage and deterioration of *H. illucens* meal exacerbate physiological stress and diminish immune capacity in *M. nipponense* , consistent with the research findings in juvenile *E. coioides* (Lin et al. 2021).

The freshness of feed protein also significantly affects the antioxidant capacity and immune function of *M. nipponense* , as manifested by changes in AST, ALT, LZM, and SOD activities (Liu et al. 2010). Chang (2025) discovered that fish meal with moderate to high spoilage levels led to a notable increase in aspartate aminotransferase (AST) and alanine aminotransferase (ALT) activities in the hemolymph of *M. rosenbergii* , while superoxide dismutase (SOD) activity in the hepatopancreas was significantly reduced. According to Li, Zeng, et al. (2023), extending the storage duration of fish meal at ambient temperature significantly elevated the TVB‐N content, which subsequently caused a significant decrease in SOD and GSH‐PX activities in the hepatopancreas of *L. vannamei* . Additionally, Wu et al. (2025) reported that when shrimp meal was partially substituted for fish meal, the freshness of the shrimp meal notably diminished SOD activity in *M. rosenbergii* . In the current study, it was observed that feeding *M. nipponense* with compound diets containing moderately and highly decomposed degreased *H. illucens* meal resulted in significantly lower hepatopancreatic SOD activity compared to the fresh degreased *H. illucens* meal control group. Conversely, AST, ALT, and LZM activities in the hemolymph were significantly elevated compared to the control group. This suggests that the spoilage level of degreased *H. illucens* meal impaired the non‐specific immune capacity of *M. nipponense* . The marked increase in ACP, ALP, and GSH‐PX activities, along with MDA content in the hemolymph of *M. nipponense* , highlights that the spoilage and deterioration of *H. illucens* meal exacerbate physiological stress and diminish immune capacity in *M. nipponense* , consistent with the research findings in juvenile *E. coioides* (Lin et al. 2021).

The hepatopancreas is a key organ that determines the digestive and absorptive capacity of shrimp (Song et al. 2013). The digestive enzymes of aquatic crustaceans include proteases, lipases, and amylases (Wu et al. 2025). The activity of hepatopancreatic digestive enzymes determines the digestive and assimilative capacity of shrimp for nutrients at the microscopic level and macroscopically manifests as the efficiency of feed utilization by shrimp (Furné et al. 2005; Bolasina et al. 2006). Research has investigated the impact of substituting fish meal with insect protein on the activity of digestive enzymes in various aquatic species (Li et al. 2017). In the case of *Ctenopharyngodon idella* , replacing fish meal with degreased *H. illucens* meal in their diet did not lead to notable alterations in the activities of their intestinal and hepatic digestive enzymes (Huang et al. 2019). Similarly, when *T. molitor* meal was used in suitable proportions to replace fish meal, the hepatopancreatic digestive enzyme activities in *L. vannamei* remained unchanged (Rios et al. 2021). However, an excessively high replacement ratio of housefly maggot meal for fish meal suppressed the digestive enzyme activity of *M. rosenbergii* (Cao et al. 2012). The current research determined that altering the replacement ratio of degreased *H. illucens* meal did not notably influence the amylase activity in *M. nipponense* . At a 20% replacement ratio, the trypsin (TPS) and lipase (LPS) activities were significantly enhanced, possibly because the degreased *H. illucens* meal has a high protein content and still retains some fat.

Several factors influence the activity of digestive enzymes in shrimp, with the freshness of dietary protein playing a decisive role. α‐amylase (α‐AMS), lipase (LPS), and trypsin (TPS) are key enzymes for evaluating the digestive function of crustacean aquatic organisms; changes in their activity directly reflect the digestive capacity of shrimp and indirectly reflect their feed utilization (Jiao et al. 2025). Wu et al. (2025) found that feeding *M. rosenbergii* shrimp meal of medium freshness (TVB‐N 200 mg/100 g) and low freshness (TVB‐N 300 mg/100 g) significantly reduced their hepatopancreatic lipase activity. Lin et al. (2021) showed that when low‐quality fish meal diets (TVB‐N 90, 135 mg/100 g) were used to formulate compound feed for juvenile *E. coioides* , the hepatopancreatic trypsin (TPS) activity was significantly reduced. The current research revealed that feeding *M. nipponense* with compound diets containing moderately and highly decomposed degreased *H. illucens* meal (TVB‐N 65 mg/100 g and 80 mg/100 g) led to a significant decrease in the activities of α‐AMS, LPS, and TPS in their hepatopancreas. Previous studies have indicated a positive correlation between dietary protein digestibility and the quality of fish meal in feed, where superior fish meal quality results in improved apparent protein digestibility (Mundheim et al. 2004). The marked reduction in the activities of α‐amylase (α‐AMS), trypsin (TPS), and lipase (LPS) would impair the ability of *M. nipponense* to utilize dietary starch, protein, and fat, which is reflected in an increased feed conversion ratio during cultivation, aligning with the findings of this study. Additionally, this research observed a significant increase in the activities of AST, ALT, and GSH‐PX in the hepatopancreas, along with elevated levels of AST, ALT, ACP, ALP, and LZM in the hemolymph of *M. nipponense* , and a notable rise in MDA content. This indicates that the TVB‐N in moderately and highly decomposed degreased *H. illucens* meal may have induced the hepatopancreatic stress or damage of *M. nipponense* , leading to activation of the non‐specific immune system to some extent. However, the specific physiological and biochemical processes and molecular signal transduction mechanisms require further research.

It is important to note that while the dose–response model predicted a peak SGR at 24.25% substitution, this value was not directly tested in our experimental design, and the model's *R* ^2^ (0.7147) indicates only a moderate fit. Therefore, we recommend interpreting this result as a reference trend rather than a definitive optimum. From a practical standpoint, the 20% substitution group exhibited the most consistent and significant improvements across multiple parameters (growth, feed efficiency, nutrient deposition, and immunity), with no additional benefits observed at higher inclusion levels. Thus, we propose an optimal substitution range of 20%–25% for practical feed formulation. Future studies employing finer gradient designs are warranted to precisely determine the exact optimum and to validate the model prediction under commercial farming conditions.

## 5 Conclusion

Replacing 20% of the fish meal with degreased *H. illucens* meal resulted in significantly enhanced growth performance, nutrient content, and immune function in *M. nipponense* compared to the control group receiving whole fish meal. In contrast, the use of moderately and highly decomposed degreased *H. illucens* meal had a significantly negative impact on the growth performance, nutrient content, digestive capacity, and immune function of *M. nipponense* . Therefore, when using degreased *H. illucens* meal to replace fish meal in formulating feed for *M. nipponense* , fresh degreased *H. illucens* meal should be used, and the replacement ratio is recommended to be within the range of 20%–25%. The model‐predicted peak near 24.25% supports this range, but direct experimental validation with finer substitution gradients is needed to confirm the exact optimal level in future.

## Author Contributions

**Huang Xin:** formal analysis, investigation, methodology. **Sheng Yin Wang:** resources, funding acquisition, writing – original draft, writing – review and editing, methodology, investigation, project administration. **Xing Er Luo:** resources. **Huang Yangdi:** conceptualization, software, methodology. **Jing Sha:** investigation, visualization, formal analysis. **Xue Bin:** validation, visualization, formal analysis, project administration. **Wu Guo Hua:** funding acquisition, project administration, resources. **Wei Shen:** investigation, formal analysis, supervision. **Yang Leyan:** project administration, validation. **Li Danyang:** visualization, software.

## Funding

This work was supported by the Zhejiang Provincial Natural Science Foundation (ZCLJHSZ26C1701), Scientific Research Startup Fund of Xingzhi College, Zhejiang Normal University (ZC302925001), and Livelihood Science and Technology Innovation Research Project in Jiaxing City (2023AZ31006, 2024AZ30007, and 2025CGZ009), 2026 College Students’ Innovation and Entrepreneurship Training Program Project (7).

## Conflicts of Interest

The authors declare no conflicts of interest.

## Data Availability

The data that support the findings of this study are available on request from the corresponding author.

## References

[fsn372274-bib-0001] Aksnes, A. , and H. Mundheim . 1997. “The Impact of Raw Material Freshness and Processing Temperature for Fish Meal on Growth, Feed Efficiency and Chemical Composition of Atlantic Halibut ( *Hippoglossus hippoglossus* ).” Aquaculture 149: 87–106. 10.1016/S0044-8486(96)01438-X.

[fsn372274-bib-0002] Albrektsen, S. , H. Mundheim , and A. Aksnes . 2006. “Growth, Feed Efficiency, Digestibility and Nutrient Distribution in Atlantic Cod ( *Gadus morhua* ) Fed Two Different Fish Meal Qualities at Three Dietary Levels of Vegetable Protein Sources.” Aquaculture 261: 626–640. 10.1016/j.aquaculture.2006.08.031.

[fsn372274-bib-0003] Bekhit, A. E. D. A. , S. G. Giteru , B. W. B. Holman , et al. 2021. “Total Volatile Basic Nitrogen and Trimethylamine in Muscle Foods: Potential Formation Pathways and Effects on Human Health.” Comprehensive Reviews in Food Science and Food Safety 20: 3620–3666. 10.1111/1541-4337.12764. 34056832

[fsn372274-bib-0004] Bekhit, A. E. D. A. , B. W. B. Holman , S. G. Giteru , et al. 2021. “Total Volatile Basic Nitrogen (TVB‐N) and Its Role in Meat Spoilage: A Review.” Trends in Food Science & Technology 109: 280–302. 10.1016/J.TIFS.2021.01.006.

[fsn372274-bib-0005] Bolasina, S. , M. Tagawa , Y. Yamashita , et al. 2006. “Effect of Stocking Density on Growth, Digestive Enzyme Activity and Cortisol Level in Larvae and Juveniles of Japanese Flounder, *Paralichthys olivaceus* .” Aquaculture 259: 432–443. 10.1016/j.aquaculture.2006.05.021.

[fsn372274-bib-0006] Cai, Y. W. , H. F. Huang , W. X. Yao , et al. 2022. “Effects of Fish Meal Replacement by Three Protein Sources on Physical Pellet Quality and Growth Performance of Pacific White Shrimp ( *Litopenaeus vannamei* ).” Aquaculture Reports 25: 101210. 10.1016/J.AQREP.2022.101210.

[fsn372274-bib-0007] Cao, J. M. , J. Yan , G. X. Wang , et al. 2012. “Effects of Replacement of Fish Meal With Housefly Maggot Meal on Digestive Enzymes, Transaminases Activities and Hepatopancreas Histological Structure of *Litopenaeus vannamei* .” South China Fisheries Science 5: 72–79. 10.3969/j.issn.2095-0780.2012.05.011.

[fsn372274-bib-0073] Chang, E. H. 2025. “Effects of Dietary Protein Levels and Freshness of Protein Sources on the Growth Performance and Immune Response of *Macrobrachium rosenbergii*.” Yangzhou University. 10.27441/d.cnki.gyzdu.2025.002195.

[fsn372274-bib-0008] Chen, H. Z. , M. Zhang , B. Bhandari , et al. 2019. “Development of a Novel Colorimetric Food Package Label for Monitoring Lean Pork Freshness.” LWT‐ Food Science and Technology 99: 43–49. 10.1016/j.lwt.2018.09.048.

[fsn372274-bib-0009] Chen, J. Z. , X. L. Zang , S. L. Meng , et al. 2012. “Effect of Nitrite Nitrogen Stress on the Activities of Nonspecific Immune Enzymes in Serum of Tilapia (GIFT *Oreochromis niloticus* ).” Ecology and Environmental Sciences 5: 897–901. 10.3969/j.issn.1674-5906.2012.05.017.

[fsn372274-bib-0010] Chen, L. , J. X. Xu , L. J. Li , C. F. Zhao , and X. W. Long . 2023. “Effects of Dietary Fishmeal Replacement by *Periplaneta americana* Meal on Biochemical Indexes, Disease Resistance and Metabolomics of Juvenile *Oncorhynchus mykiss* .” South China Fisheries Science 4: 86–97. 10.12131/20220208.

[fsn372274-bib-0011] Chen, Y. K. , C. Z. Zheng , S. Zhang , et al. 2023. “Dietary Black Soldier Fly ( *Hermetia illucens* ) Larvae Meal on Growth Performance, Non‐Specific Immunity and Lipid Metabolism of *Litopenaeus vannamei* .” Acta Hydrobiologica Sinica 2: 269–278. 10.7541/2023.2022.0201.

[fsn372274-bib-0012] Chen, Z. Y. , Q. X. Li , R. M. Sheng , et al. 2025. “Effects of Defatted Black Soldier Fly Larvae Meal on Growth, Nutrient Digestibility, Hepatopancreas Biochemistry, Intestinal Microbiota, and Phosphorus Discharge of Giant Freshwater Prawn ( *Macrobrachium rosenbergii* ).” Animal Nutrition 23: 271–285. 10.1016/j.aninu.2025.08.001. 41321527 PMC12664078

[fsn372274-bib-0013] Cheng, H. H. , C. X. Xie , D. P. Li , et al. 2016. “The Study of Muscular Nutritional Components and Fish Quality of Grass Carp ( *Ctenopharyngodon idella* ) in Ecological Model of Cultivating Grass Carp With Grass.” Journal of Fisheries of China 7: 1050–1059. 10.11964/jfc.20150709964.

[fsn372274-bib-0014] Ding, J. Q. , P. Liu , J. Li , et al. 2013. “Comparison of Nonspecific Immunity and the Activities of Antioxidant Enzymes in Different Populations of *Charybdis japonica* .” Journal of Fisheries of China 2: 275–280. 10.3724/SP.J.1231.2013.38145.

[fsn372274-bib-0015] Ettefaghdoost, M. , and H. Haghighi . 2025. “The Growth‐Stimulatory, Immunomodulatory, Antioxidative, and Metabolic Regulation Impacts of Dietary Chitosan Supplementation on the Oriental River Prawn ( *Macrobrachium nipponense* ).” Aquaculture 608: 742771. 10.1016/j.aquaculture.2025.742771.

[fsn372274-bib-0016] Fischer, H. , N. Romano , N. Renukdas , et al. 2022. “Comparing Black Soldier Fly (*Hermetia illucens*) Larvae Versus Prepupae in the Diets of Largemouth Bass, *Micropterus salmoides*: Effects on Their Growth, Biochemical Composition, Histopathology, and Gene Expression.” Aquaculture 546: 737323. 10.1016/J.AQUACULTURE.2021.737323.

[fsn372274-bib-0017] Furné, M. , M. C. Hidalgo , A. López , et al. 2005. “Digestive Enzyme Activities in Adriatic Sturgeon *Acipenser naccarii* and Rainbow Trout *Oncorhynchus mykiss* . A Comparative Study.” Aqua‐Culture 250: 391–398. 10.1016/j.aquaculture.2005.05.017.

[fsn372274-bib-0018] Galán Díaz, J. J. , L. P. Mosquera , J. P. Agudo , and J. Rodríguez . 2026. “Carbon and Water Footprint Assessment of the Production Cycle of the Black Soldier Fly ( *Hermetia illucens* ) on a Farm in Spain.” Environmental Development 51: 101038. 10.1016/J.ENVDEV.2024.101038.

[fsn372274-bib-0019] Han, G. M. , J. H. Jia , L. M. Lei , et al. 2023. “Effects of Replacing Fish Meal With Black Soldier Fly Larvae Meal on Growth, Body Composition and Immunity of Red Swamp Crayfish ( *Procambarus clarkii* ).” Chinese Journal of Animal Nutrition 35, no. 7: 4495–5606. 10.12418/CJAN2023.418.

[fsn372274-bib-0020] Hu, L. , B. Yun , M. Xue , et al. 2013. “Effects of Fish Meal Quality and Fish Meal Substitution by Animal Protein Blend on Growth Performance, Flesh Quality and Liver Histology of Japanese Seabass ( *Lateolabrax japonicus* ).” Aquaculture 372–375: 52–61. 10.1016/j.aquaculture.2012.10.025.

[fsn372274-bib-0021] Huang, W. Q. , Y. H. Huang , H. F. Mi , et al. 2019. “Effects of Fishmeal Replacement by Three Animal Protein Sources on Growth Performance, Muscle Nutritional Composition, Digestive Enzyme Activities, Serum Biochemical and Antioxidant Indices of *Ctenopharyngodon idella* .” Chinese Journal of Animal Nutrition 5: 2187–2200. 10.3969/j.issn.1006-267x.2019.05.026.

[fsn372274-bib-0022] Huang, X. X. , C. X. Luo , T. F. Guo , et al. 2012. “Toll Receptor in Prawn and Its Application in Nutrition‐Immunity Assessing on Prawn.” Journal of Fisheries of China 6: 930–936. 10.3724/SP.J.1231.2012.27774.

[fsn372274-bib-0023] Jeong, S.‐M. , S. Khosravi , I. R. Mauliasari , et al. 2020. “Dietary Inclusion of Mealworm ( *Tenebrio molitor* ) Meal as an Alternative Protein Source in Practical Diets for Rainbow Trout ( *Oncorhynchus mykiss* ) Fry.” Fisheries and Aquatic Sciences 23: 1–8. 10.1186/s41240-020-00158-7.

[fsn372274-bib-0024] Jiang, L. N. , J. Y. Tang , F. Y. Huang , et al. 2025. “Comparative Study on Muscle Nutritional Composition and Nutritional Value Between the Introduced Species of *Macrobrachium nipponense* Taihu Lake 2 and the Native Species Under the Rice‐Shrimp Co‐Culture Model.” Journal of Zhejiang Agricultural Sciences 8: 1819–1823. 10.16178/j.issn.0528-9017.20250374.

[fsn372274-bib-0025] Jiang, L. Y. , Y. Y. Xu , T. Z. Zhou , et al. 2026. “Effects of Black Soldier Fly Larvae Meal on Growth Performance, Immune Function, and Serum Parameters in Yellow‐Feathered Broilers.” Feed Research 3: 49–54. 10.13557/j.cnki.issn1002-2813.2026.03.010.

[fsn372274-bib-0026] Jiao, T. , Z. W. Xu , Y. X. Cai , and Z. M. Gu . 2025. “Effects of Dietary Fermentation Products of Table Leftovers on Growth, Muscle Quality, Immune Enzyme Activity and Digestive Function for *Procambarus clarkii* .” Journal of Huazhong Agricultural University 1: 191–200. 10.13300/j.cnki.hnlkxb.2025.01.021.

[fsn372274-bib-0027] Li, L. C. , J. L. Xie , H. X. Huang , and Q. M. Zhang . 2024. “Feasibility Analysis of Rapid Detection of Lead‐Cadmium in Base Shrimp.” Journal of Food Safety and Quality 15: 316–322. 10.19812/j.cnki.jfsq11-5956/ts.20240303005.

[fsn372274-bib-0028] Li, S. , H. Ji , B. Zhang , et al. 2017. “Degreased Black Soldier Fly ( *Hermetia illucens* ) Larvae Meal in Diets for Juvenile Jian Carp ( *Cyprinus carpio* var. Jian): Growth Performance, Antioxidant Enzyme Activities, Digestive Enzyme Activities, Intestine and Hepatopancreas Histological Structure.” Aquaculture 477: 62–70. 10.1016/j.aquaculture.2017.04.015.

[fsn372274-bib-0029] Li, Y. , Y. Y. Zeng , Q. F. Liu , et al. 2023. “Characteristics of Different Submerged Macrophytes on the Growth and Survival of *Macrobranchium nipponense* and Their Effects on Aquatic Environment.” Chinese Fishery Quality and Standards 13: 1–10. 10.3969/j.issn.2095-1833.2023.06.001.

[fsn372274-bib-0030] Li, Z. Z. , B. Liu , Z. H. Liu , et al. 2023. “Fermented Feed on Growth Performance, Antioxidant Capacity and Intestinal Microorganisms of *Macrobrachium nipponense* .” Acta Hydrobiologica Sinica 9: 1435–1445. 10.7541/2023.2022.0496.

[fsn372274-bib-0031] Liang, Q. H. , C. Li , B. P. Tan , et al. 2026. “Effects of Replacement of Inorganic Trace Elements by Organic Trace Elements in Alternative Diets of *Tenebrio molitor* on Growth Performance, Antioxidant Capacity, Immunity and Intestinal Flora of *Litopenaeus vannamei* .” Aquaculture Reports 46: 103361. 10.1016/j.aqrep.2026.103361.

[fsn372274-bib-0032] Lin, H. X. , Q. H. Yang , A. J. Wang , et al. 2021. “Effects of Fish Meal Under Different Storage Conditions on Growth, Serum Biochemical Indices and Antioxidant Capacity for Juvenile Grouper *Epinephelus coioides* .” Aquaculture Nutrition 27: 723–733. 10.1111/anu.13218.

[fsn372274-bib-0033] Liu, B. , J. Xie , X. Ge , et al. 2010. “Effects of Anthraquinone Extract From *Rheum officinale* Bail on the Growth Performance and Physiological Responses of *Macrobrachium rosenbergii* Under High Temperature Stress.” Fish & Shellfish Immunology 29: 49–57. 10.1016/j.fsi.2010.02.018. 20219682

[fsn372274-bib-0034] Liu, J. H. , Z. G. Hu , Y. J. Lei , et al. 2025. “Advances and Prospects in Genetics and Breeding of Freshwater Prawn *Macrobrachium nipponense* .” Fisheries Science 5: 836–844. 10.16378/j.cnki.1003-1111.24210.

[fsn372274-bib-0035] Liu, S. , Q. R. Mo , J. Wang , Y. Cui , and L. Tian . 2025. “Main Biological Characteristics of *Hermetia illucens* L. and Its Potential Applications as a Model Organism.” Laboratory Animal and Comparative Medicine 6: 803–809. 10.12300/j.issn.1674-5817.2025.141.

[fsn372274-bib-0036] Liu, Y. Y. , P. B. Brown , Z. Pei , et al. 2023. “Effects of the Replacement of Fish Meal With Cricket Meal on Growth Performance, Muscle Amino Acid Composition, and Serum Biochemical Indices of Juvenile Yellow Catfish ( *Pelteobagrus fulvidraco* ).” Progress in Fishery Sciences 5: 69–79. 10.19663/j.issn2095-9869.20230213001.

[fsn372274-bib-0072] Liu, Z. S. , X. Y. Qian , L. Y. Wei , et al. 2025. “Dietary Riboflavin Levels on the Mitochondrial Biogenesis and Function of Oriental River Prawn (*Macrobrachium nipponense*).” Acta Hydrobiologica Sinica 49: 092512. 10.3724/1000-3207.2025.2024.0452.

[fsn372274-bib-0037] Long, X. W. , Y. T. Liu , Y. Q. Li , et al. 2022. “Effects of Dietary American Cockroach *Periplaneta americana* Meal Inclusion on the Growth Performance, Antioxidant Capacity, and Immunity of Juvenile Rainbow Trout *Oncorhynchus mykiss* .” Aquaculture Nutrition 2022: 8359405. 10.1155/2022/8359405.

[fsn372274-bib-0038] Ma, L. , J. F. Jiang , H. M. Wu , et al. 2018. “Analysis and Evaluation of Nutritional Components in the Muscle of 5 Strains of *Penaeus vannamei* Cultured in Circulating Water in Ponds.” Fishery Modernization 5: 36–44. 10.3969/j.issn.1007-9580.2018.05.007.

[fsn372274-bib-0039] Ma, L. Q. , C. L. Qi , J. J. Cao , and D. P. Li . 2014. “Comparative Study on Muscle Texture Profile and Nutritional Value of Channel Catfish ( *Ictalurus punctatus* ) Reared in Ponds and Reservoir Cages.” Journal of Fisheries of China 4: 531–536. 10.3724/SP.J.1231.2014.48963.

[fsn372274-bib-0040] Mkilima, T. , M. Lauwo , L. Laurah Gutu , and M. Basitere . 2026. “Optimising Environmental Conditions for Sustainable Maggot Production From Organic Waste: Influence of Temperature, Humidity, and Light on *Hermetia illucens* Growth and Bioconversion Efficiency.” Waste and Biomass Valorization. 10.1007/S12649-026-03693-X.

[fsn372274-bib-0041] Moksness, E. , G. Rosenlund , and A. Lie . 2008. “Effect of Fish Meal Quality on Growth of Juvenile Wolffish, *Anarhichas lupus* L.” Aquaculture Research 26: 109–115. 10.1111/j.1365-2109.1995.tb00890.x.

[fsn372274-bib-0042] Mundheim, H. , A. Aksnes , and B. Hope . 2004. “Growth, Feed Efficiency and Diges‐Tibility in Salmon ( *Salmo salar* L.) Fed Different Dietary Proportions of Vegetable Protein Sources in Combination With Two Fish Meal Qualities.” Aquaculture 237: 315–331. 10.1016/j.aquaculture.2004.03.011.

[fsn372274-bib-0043] Qian, X. Y. , X. F. Li , L. Zhang , et al. 2023. “Effects of Dietary Riboflavin Levels on the Growth Performance and Metabolic Function of *Macrobrachium nipponense* .” Journal of Fisheries of China 10: 197–209. 10.11964/jfc.20230714066.

[fsn372274-bib-0044] Renna, M. , A. Schiavone , F. Gai , et al. 2017. “Evaluation of the Suitability of a Partially Degreased Black Soldier Fly ( *Hermetia illucens* L.) Larvae Meal as Ingredient for Rainbow Trout ( *Oncorhynchus mykiss* Walbaum) Diets.” Journal of Animal Science and Biotechnology 8: 57. 10.1186/s40104-017-0191-3. 28680591 PMC5494141

[fsn372274-bib-0045] Ricque‐Marie, D. , M. A. D. Parra , L. E. Cruz‐Suarez , et al. 1998. “Raw Material Freshness, a Quality Criterion for Fish Meal Fed to Shrimp.” Aquaculture 165: 95–109. 10.1016/S0044-8486(98)00229-4.

[fsn372274-bib-0046] Rios, C. , R. L. Panini , L. A. A. Menezes , et al. 2021. “Effects of the Substitution of Fishmeal With Mealworm Meal on Enzymes, Haemolymph and Intestinal Microbiota of the Pacific White Shrimp.” Journal of Insects as Food and Feed 477: 62–70. 10.3920/JIFF2020.0148.

[fsn372274-bib-0047] Servillo, L. , N. D'Onofrio , A. Giovane , et al. 2018. “Ruminant Meat and Milk Contain δ‐Valerobetaine, Another Precursor of Trimethylamine N‐Oxide (TMAO) Like γ‐Butyrobetaine.” Food Chemistry 260: 193–199. 10.1016/j.food-chem.2018.03.114. 29699662

[fsn372274-bib-0048] Shi, D. J. , Y. J. Liang , Y. S. Sun , and Z. Y. Zhu . 2015. “Effects of Fish Meal Replacement by Housefly Maggot Meal on Growth, Muscle Composition and Liver Non‐Specific Immune Indices of Tilapia ( *Oreochromis niloticus* ).” Fisheries Science & Technology Information 6: 319–323. 10.16446/j.cnki.1001-1994.2015.06.006.

[fsn372274-bib-0049] Song, X. N. , J. F. Ma , Y. J. Qin , et al. 2013. “Effects of Abrupt Decline in Salinity on the Antioxidant Enzyme Activities and Histological Structure in *Ruditapes philippinarum* .” Journal of Agriculture 1: 50–56+70. 10.3969/j.issn.1007-7774.2013.01.011.

[fsn372274-bib-0050] Tang, J. Y. , J. Y. Ye , Y. X. Dai , A. L. Jiang , and C. Wu . 2021. “Comparison of Muscle Nutritional Compositions of Four Wild Oriental River Prawn *Macrobrachium nipponense* Populations in Jiangsu Province.” Acta Hydrobiologica Sinica 4: 801–808. 10.7541/2021.2020.102.

[fsn372274-bib-0051] Tantikitti, C. , D. Chookird , and A. Phongdara . 2016. “Effects of Fishmeal Quality on Growth Performance, Protein Digestibility and Trypsin Gene Expression in Pacific White Shrimp ( *Litopenaeus vannamei* ).” Songklanakarin Journal of Science and Technology 38: 73–82.

[fsn372274-bib-0052] Vasagam, K. P. K. , S. Ramesh , and T. Balasubramanian . 2005. “Dietary Value of Different Vegetable Oil in Black Tiger Shrimp *Penaeus monodon* in the Presence and Absence of Soy Lecithin Supplementation: Effect on Growth, Nutrient Digestibility and Body Composition.” Aquaculture 250: 317–327. 10.1016/j.aquaculture.2005.02.035.

[fsn372274-bib-0053] Wang, A. R. , J. Xu , X. Zhang , et al. 2026. “Effects of the Fishmeal Freshness on Growth Performance, Immune Response, Hepatopancreatic Health and Anti‐Stress Capacity of Giant Freshwater Prawn ( *Macrobrachium rosenbergii* ).” Aquaculture Reports 46: 103281. 10.1016/j.aqrep.2025.103281.

[fsn372274-bib-0054] Wang, B. B. , X. M. Ding , M. M. Cai , et al. 2026. “Progress on Black Soldier Fly as an Alternative Source of Protein for Feed.” Animal Breeding and Feed 1: 7–14. 10.13300/j.cnki.cn42-1648/s.2026.01.002.

[fsn372274-bib-0055] Wang, N. , Y. Zheng , Z. P. Liu , et al. 2023. “Effects of Three Inputs on Antioxidant Enzymes, MDA, Phosphatase and LZM of *Procambarus clarkii* .” Freshwater Fisheries 3: 87–92. 10.3969/j.issn.1000-6907.2023.03.011.

[fsn372274-bib-0056] Weng, X. Z. , X. Shao , J. Q. Ye , et al. 2024. “Effects of Replacing Fish Meal With Degreased Black Soldier Fly Larvae Meal on Growth Performance and Muscle Quality of Largemouth Bass ( *Micropterus salmoides* ).” Feed Research 24: 70–74. 10.13557/j.cnki.issn1002-2813.2024.24.014.

[fsn372274-bib-0057] Worlanyo, H. G. , S. F. Jiang , Y. B. Yu , et al. 2022. “Effects of Dietary Threonine on Growth and Immune Response of Oriental River Prawn ( *Macrobrachium nipponense* ).” Fish & Shellfish Immunology 128: 288–299. 10.1016/J.FSI.2022.07.072. 35921934

[fsn372274-bib-0058] Wu, X. , G. Chang , Y. Cheng , et al. 2010. “Effects of Dietary Phospholipid and Highly Unsaturated Fatty Acid on the Gonadal Development, Tissue Proximate Composition, Lipid Class and Fatty Acid Composition of Precocious Chinese Mitten Crab, *Eriocheir sinensis* .” Aquaculture Nutrition 16: 25–36. 10.1111/j.1365-2095.2008.00637.x.

[fsn372274-bib-0059] Wu, Y. H. , J. Xu , K. Y. Deng , et al. 2025. “Effects of Shrimp Meal Freshness on Growth Performance, Antioxidant Capacity and Hepatopancreas Health of *Macrobrachium rosenbergii* .” Chinese Journal of Animal Nutrition 11: 7807–7819. 10.12418/CJAN2025.634.

[fsn372274-bib-0060] Xu, H. , J. B. Wang , T. L. Xu , et al. 2026. “Effects of Sodium Butyrate Supplementation on Growth, Immune Response, Hepatopancreatic Morphology and mRNA Expre‐Ssion of Genes Related to Insulin Signaling Pathway of *Macrobrachium nipponense* .” Feed Industry 2: 111–118. 10.13302/j.cnki.fi.2026.02.015.

[fsn372274-bib-0061] Xu, J. , K. Y. Deng , E. H. Chang , et al. 2024. “Effects of Freshness of Poultry By‐Product Meal on the Growth Performance, Immune Response, and Hepatopancreatic Health of *Macrobrachium rosenbergii* .” Aquaculture Reports 39: 102443. 10.1016/j.aqrep.2024.102443.

[fsn372274-bib-0062] Yu, W. J. , Y. Zhang , W. W. Zeng , L. F. Luo , and J. P. Zhang . 2023. “Effect of Aqueous Extract of *Periplaneta americana* on Growth Performance, Serum and Liver Bio‐Chemical Indices in *Oreochromis niloticus* .” Feed Research 1: 70–74. 10.13557/j.cnki.issn1002-2813.2023.01.015.

[fsn372274-bib-0063] Zhang, H. M. 2024. “Application of *Periplaneta americana* Meal Instead of Fishmeal in the Feed of *Macrobrachium rosenbergii* .” M.S. Thesis. Guangdong Ocean University. 10.27788/d.cnki.ggdhy.2024.000038.

[fsn372274-bib-0064] Zhang, L. , X. Q. Liu , B. Yun , et al. 2024. “Effects of Black Fly Larvae Meal on Growth, Immunity, and Antioxidant Performance of *Macrobrachium rosenbergii* Juveniles.” Feed Research 4: 63–67. 10.13557/j.cnki.issn1002-2813.2024.04.012.

[fsn372274-bib-0065] Zhang, L. , J. Zhou , H. Y. Ke , et al. 2024. “Genetic Diversity of *Macrobrachium nipponense* Populations in Different River Basins in Sichuan Province.” Southwest China Journal of Agricultural Sciences 37: 1124–1130. 10.16213/j.cnki.scjas.2024.5.025.

[fsn372274-bib-0074] Zhang, W. H. , W. W. Wang , H. K. Wang , et al. 2026. “Nutritional Value of Black Soldier Fly and Its Application in Pet Diets.” Feed Research 49: 164–169. 10.13557/j.cnki.issn1002-2813.2026.07.028.

[fsn372274-bib-0066] Zhao, Z. M. , W. X. Wu , D. Zhou , Z. H. Li , and Y. Q. Kong . 2025. “Different Sources of Dietary Chromium on Growth, Immunity, and Glucose and Lipid Metabolism of *Macrobrachium nipponense* .” Acta Hydrobiologica Sinica 9: 130–141. 10.3724/1000-3207.2025.2025.0025.

[fsn372274-bib-0067] Zheng, J. X. , D. S. Zhou , S. S. Wei , et al. 2022. “Effects of Animal and Plant Proteins on Growth, Muscle Composition, Antioxidant Abilities, mRNA Expressions of Genes Related to TOR Signaling Pathway and Appetite Regulation in *Macrobrachium nipponense* .” Journal of Fisheries of China 10: 1801–1812. 10.11964/jfc.20220613561.

[fsn372274-bib-0068] Zhou, J. S. , Y. S. Chen , H. Ji , and E. M. Yu . 2017. “The Effect of Replacing Fish Meal With Fermented Meal Mixture of Silkworm Pupae, Rapeseed and Wheat on Growth, Body Composition and Health of Mirror Carp ( *Cyprinus carpio* var. Specularis).” Aquaculture Nutrition 23: 741–754. 10.1111/anu.12441.

[fsn372274-bib-0069] Zhu, J. C. , Y. N. Sun , Y. X. Yang , et al. 2022. “Muscle Biochemical Compositions and Dietary Characteristics Based on Characteristic Fatty Acids of *Exopalaemon modestus* and *Macrobrachium nipponense* in Chaohu Lake.” Freshwater Fisheries 5: 25–31. 10.13721/j.cnki.dsyy.2022.05.013.

[fsn372274-bib-0070] Zoli, M. , I. Guerreiro , J. Bacenetti , S. B. Oddon , and P. Enes . 2026. “Towards Cleaner Aquaculture: Assessing the Environmental Performance of Meagre ( *Argyrosomus regius* ) Fed With Insect Meal ( *Hermetia illucens* ).” Journal of Cleaner Production 565: 148578. 10.1016/J.JCLEPRO.2026.148578.

[fsn372274-bib-0071] Zubair, A. A. 2026. “Beyond Overfishing: Environmental Footprints of Modern Fishing Practices and Their Implications for Ocean Health.” International Journal of Environment and Climate Change 16: 85–106. 10.9734/IJECC/2026/V16I25268.

